# Degradation Determinants Are Abundant in Human Noncanonical Proteins and Minor Annotated Isoforms

**DOI:** 10.1093/gbe/evag009

**Published:** 2026-01-19

**Authors:** Claudio Casola, Adekola Owoyemi, Nikolaos Vakirlis

**Affiliations:** Department of Ecology and Conservation Biology, Texas A&M University, 534 John Kimbrough Blvd, TAMU 2258, College Station, TX 77845-2258, USA; Interdisciplinary Doctoral Degree Program in Ecology and Evolutionary Biology, Texas A&M University, College Station, TX 77845, USA; Interdisciplinary Doctoral Degree Program in Genetics and Genomics, Texas A&M University, College Station, TX 77845, USA; Department of Ecology and Conservation Biology, Texas A&M University, 534 John Kimbrough Blvd, TAMU 2258, College Station, TX 77845-2258, USA; Hellenic Pasteur Institute, 127 Vas. Sofias Ave, Athens 11521, Greece

**Keywords:** noncanonical ORFs, microproteins, cryptic proteins, proteasome, protein degradation

## Abstract

The comprehensive characterization of human proteins, a key objective in contemporary biology, has been revolutionized by the identification of thousands of potential novel proteins through ribosome profiling and proteomics. Determining the physiological activity of these noncanonical proteins has proven difficult, because they are encoded by different types of coding regions and tend to share no sequence similarity with canonical polypeptides. Evidence from immunopeptidomic assays combined with a better understanding of the quality control of protein synthesis suggest that many noncanonical proteins may possess low stability in the cellular environment. Here, we tested this hypothesis by analyzing the frequency of multiple sequence features eliciting either proteasomal degradation or autophagy across 91,003 canonical (annotated) protein isoforms and 11,499 noncanonical proteins. Overall, noncanonical proteins were enriched for degradation-related features compared to all canonical proteins. Notably, degradation determinants were also enriched in canonical protein isoforms starting with a non-methionine amino acid. Analyses of original and shuffled sequences showed evidence of selective pressure either against or toward the accumulation of specific degradation signatures only in major isoforms of canonical proteins. However, stability was significantly higher in noncanonical proteins with evidence of phenotypic effects upon knock-out in cell lines. Notably, we found that the C-terminal tail hydrophobicity represents a reliable proxy for degradation propensity with potential applications in identifying functional noncanonical proteins. These findings underscore the critical role of degradation processes in regulating the half-life of noncanonical proteins and demonstrate the power of degradation-associated signatures in discriminating noncanonical genes likely to encode biologically functional molecules.

Significance Recent advances have uncovered thousands of previously unknown human proteins, but determining which of these are biologically meaningful remains a major challenge. This study reveals that many of these noncanonical proteins are marked for rapid degradation, suggesting that many of them are typically unstable. By identifying specific sequence features that predict protein stability, this work offers a new approach to distinguish potentially important proteins from likely byproducts of gene expression.

## Introduction

Annotating all proteins encoded by the human genome is paramount to our understanding of human physiology, disease and evolution. The number of known, or “canonical,” protein coding genes has been shown to be around 20,000, with many genes encoding multiple protein isoforms derived from alternative splicing (Pruitt et al. 2009; Tapial et al. 2017; Abascal et al. 2018). However, it is becoming increasingly appreciated that human genome harbors many noncanonical open reading frames (ncORFs) with the potential to encode polypeptides (Basrai et al. 1997; Frith et al. 2006; Calvo et al. 2009; Brunet et al. 2018). Typically, ncORFs are defined as translated ORFs located outside or on an alternative frame of the annotated protein-coding regions of a transcript. Noncanonical ORFs also include ORFs initiating from non-AUG codons within annotated transcripts (Mudge et al. 2022). Noncanonical ORFs tend to be significantly shorter than canonical coding sequences, thus disproportionally encoding small proteins that have largely been overlooked by gene annotation algorithms. Collectively, these proteins are sometimes referred to as the “dark proteome” (Couso and Patraquim 2017; Ruiz-Orera and Alba 2019).

The development of ribosome profiling (Ribo-seq) technologies has dramatically increased the ability to identify potentially translated ncORFs (Ingolia et al. 2009; Mackowiak et al. 2015; Raj et al. 2016; Samandi et al. 2017; Lu et al. 2019; van Heesch et al. 2019; Chen et al. 2020; Chong et al. 2020; Cuevas et al. 2021; Prensner et al. 2021; Chothani et al. 2022; Ouspenskaia et al. 2022). Additionally, proteomic data have directly showed synthesis of noncanonical proteins (Slavoff et al. 2013; Vanderperre et al. 2013; Kim et al. 2014; Lu et al. 2019), and evolutionary analyses revealed selective constraints compatible with protein-coding sequences in a subset of ncORFs (Ladoukakis et al. 2011; Mackowiak et al. 2015).

However, a physiological role has been conclusively demonstrated only for a limited number of noncanonical proteins, or NCPs, raising the question of how many of these sequences constitute functional polypeptides. Furthermore, estimates of these proteins spans three orders of magnitude (Hao et al. 2018; Brunet et al. 2021; Mudge et al. 2022), and there is limited overlap in NCPs prediction between programs used to analyzed Ribo-seq data (Chothani et al. 2023; Tong et al. 2024) and between catalogs of potentially functional NCPs detected using CRISPR/Cas9 knock-out experiments (Azam et al. 2025).

Proteomic evidence also suggests that many NCPs might represent aberrant translation products that are rapidly degraded. Analyses of mass-spectrometry spectra showed that a limited number of NCPs are supported by proteomic data (Deutsch et al. 2024; Wacholder et al. 2025). Furthermore, NCPs tend to be disproportionally represented in the immunopeptidome—the collection of peptides from proteasomal cleavage that are presented by the MHC I—compared to the whole-proteasome (Laumont et al. 2018; Chong et al. 2020; Cuevas et al. 2021; Ouspenskaia et al. 2022). Overall, these results are in agreement with the “defective ribosomal products” (DRiPs) hypothesis, which states that peptides presented by the MHC I largely derive from misfolded and prematurely terminated proteins that are rapidly degraded after their synthesis (Yewdell et al. 1996; Schubert et al. 2000). Although the DRiPs hypothesis was originally conceived to explain the rapid presentation of viral antigen upon cellular infection (Yewdell et al. 1996), it provides a useful framework to interpret the role of stability in the biological activity of noncanonical proteins.

Potential pathways that might affect NCP stability and promote their removal after translation have begun to emerge. For instance, a recent seminal study has revealed that NCPs on average possess higher hydrophobicity at the C-terminal tail than canonical proteins (hereafter, CPs), a feature associated with proteasomal-mediated proteolysis (Kesner et al. 2023). Additionally, several types of ncORFs detected via Ribo-seq might not result in a functional protein product. Indeed, some upstream ORFs (uORFs) regulate translation of the main ORF on the same transcript by interacting with ribosomes without producing a protein (Barbosa et al. 2013).

Nevertheless, there is increasing support for the view that many human noncanonical proteins are functional (Azam et al. 2025). Several physiologically important NCPs include sarcolipin (Odermatt et al. 1997), phospholamban (MacLennan and Kranias 2003), myoregulin (Anderson et al. 2015), and DWORF (Nelson et al. 2016), which regulate the sarcoendoplasmic reticulum calcium ATPase (SERCA) in the muscle, and mitoregulin, which is expressed in mitochondria where is involved in lipid metabolism (Stein et al. 2018). More broadly, knock-out experiments have shown that thousands of ncORFs exhibit a phenotypic effect, albeit in cell cultures (Chen et al. 2020; Zheng et al. 2023; Schlesinger et al. 2025), and a small but non-negligeable proportion of NCPs have been detected across multiple proteomic sequencing data (Brunet et al. 2021). A possible reason why this proportion is not higher is the difference in distribution of tryptic sites between canonical and noncanonical proteins, resulting in a high rate of NCP false negatives (Prensner et al. 2023; Wacholder and Carvunis 2023; Wacholder et al. 2025). Experimental analyses integrating Ribo-seq, mass-spectrometry, and immunopeptidomic data can further improve detection of stable NCPs (Prensner et al. 2023), although only a few such integrative approaches have been published so far (Cuevas et al. 2021; Prensner et al. 2021; Ouspenskaia et al. 2022).

Computational predictions of sequence signatures associated with protein stability can significantly contribute to determining the functional impact of NCPs, both complementing and expanding on translation surveys and on the limited number of well-characterized noncanonical proteins. For instance, stability profiles of NCPs can inform on sequences that are less likely to be rapidly degraded and therefore should be prioritized for in-depth experimental analyses. It has been shown that features, including length, presence of a domain, and evolutionary conservation tend to be associated with more stable NCPs and that the half-life of NCPs is increased upon inhibition of the ubiquitin-proteasome and autophagy pathways (Yang et al. 2024). However, it remains unclear whether specific protein sequence attributes distinguish stable NCPs from those that are more likely to be degraded.

In this study, we investigated the stability of noncanonical proteins using an array of experimentally characterized and inferred protein degradation determinants linked with either proteasomal digestion or autophagy. We analyzed 91,003 canonical proteins and 11,499 non-redundant NCPs from four Ribo-seq datasets and one meta dataset. Our results confirmed the higher C-terminal tail hydrophobicity in NCPs. We also showed a significant enrichment in noncanonical proteins of two additional categories of proteasome-related degradation determinants, end-degrons, and terminal disordered regions. These degradation signatures were less frequent in some NCP categories that are more likely to be functional, including those with a reported phenotypic effect following knock-out experiments. Intriguingly, we found no NCPs enrichment of chaperone-mediated autophagy motifs, which mediate the non-proteasomal degradation of specific proteins. Our findings reveal significant differences in the regulation of the canonical and noncanonical proteome and contribute to our understanding of the functional potential of noncanonical proteins.

## Results

### Datasets and Degradation Determinants

Human noncanonical proteins have been obtained from a variety of tissues and cell types throughout several methodological approaches. We analyzed data from four individual ribosomal profiling datasets (Ji et al. 2015; Raj et al. 2016; Chen et al. 2020; Martinez et al. 2020) and one meta-dataset (Mudge et al. 2022). The four primary studies relied on different tools to score ORFs from Ribo-seq data (Table 1). The analysis described in Raj et al (2016) employed riboHMM, which has been found to be a less reliable ORF predictor than other tools (Zhang et al. 2017). Overall, this provided the opportunity to test hypotheses on protein degradation predictions across ribo-seq data with various levels of robustness.

**Table 1:** Summary of analyzed human canonical and noncanonical protein datasets. Numbers in columns 3 to 7 show protein sequences available for analysis of different degradation determinants according to criteria described in the Methods section

Dataset	Total Proteins	CTTH	N-end degrons	C-end degrons	IDRs	CMA	Ribo-seq tool
MANE	18,802	18,443	18,130	18,117	18,453	18,802	N/A
Other-isoforms-AUG	57,720	43,834	24,637	43,053	26,326	57,720	N/A
Longest-nonAUG	3,044	3,041	Na	3,038	3,041	3,044	N/A
Other-isoforms-nonAUG	1,264	1,212	Na	1,203	1,127	1,264	N/A
Functional MPs	94	83	67	81	41	94	N/A
NCPs	11,240	10,162	5,571	10,001	4,245	11,240	N/A
Chen et al. 2020	765	727	488	719	239	765	ORF-RATER
Ji et al. 2015	4,298	4,168	2,363	4,111	1,573	4,298	RibORF
Martínez et al. 2020	3,973	3,828	2,019	3,794	1,648	3,973	RibORF
Mudge et al. 2022	4,272	4,234	4,011	4,195	1,410	4,272	N/A
Raj et al. 2016	2,475	2,379	892	2,369	918	2,475	riboHMM
iORFs	8,102	8,102	8,040	8,045	1,789	8,136	N/A

To avoid redundancy, we removed identical sequences within datasets depending on the analysis (Table 1; see also Methods). We also generated a combined set of NCPs after removing redundancy both within and across the five datasets. These NCP datasets are formed mostly by uORFs and lncRNA ORFs but also include downstream (ORFs), internal ORFs (intORFs) and ORFs within pseudogenes (Table S1). We compared NCP data with four groups of human annotated (canonical) proteins. The first group includes ∼19,000 proteoforms annotated in the Matched Annotation from NCBI and EMBL-EBI (MANE) project, or MANE dataset (Morales et al. 2022), containing highly expressed isoforms from each locus. Additionally, we analyzed ∼68,000 ENSEMBL alternative protein isoforms starting with a standard methionine (Other-isoforms-AUG), and ∼4,000 further isoforms starting with a non-methionine amino acid, further divided into Longest isoforms-nonAUG and Other-isoforms-nonAUG (Table 1; see Methods). Finally, we included in our analysis 94 sequences from microproteins that have been experimentally characterized and are considered functional (Kesner et al. 2023; Table S2). To determine the role of amino acid composition on degradation determinant frequency, we compared original protein sequences in each dataset with sequences obtained by randomly shuffling their amino acid order.

**Fig. 1.**
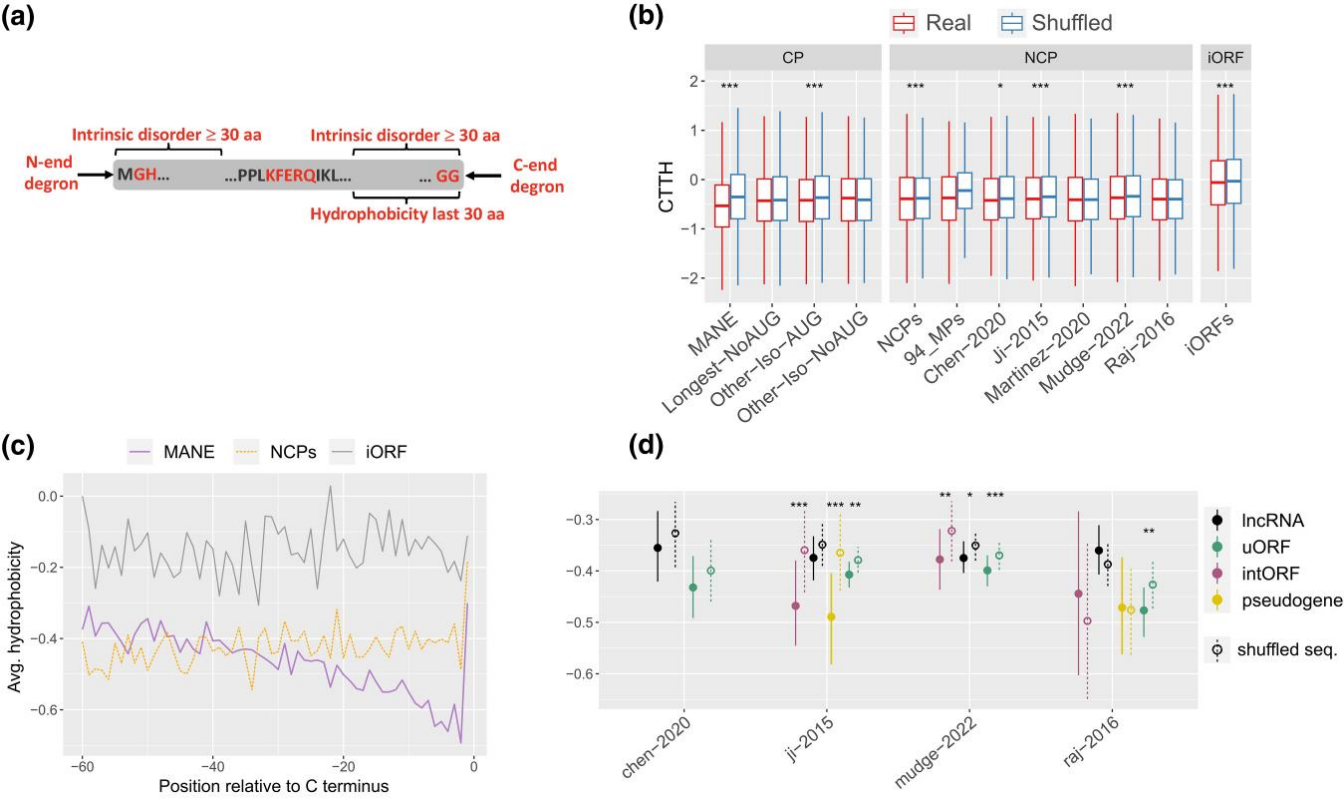
a) Representation of the four degradation determinants analyzed in this study and their localization along protein sequences along a protein sequence (gray rectangle). The N-end and C-end degron motifs correspond to two recently reported strong degrons [42–44] A KFERQ-like motif is shown around the center of the protein. b) C-terminal tail hydrophobicity scores (CTTH) calculated using the Kyte-Doolittle scale in AUG and non-AUG CPs and in NCP datasets. Values for both original (red) and shuffled (blue) sequences are shown. iORFs: nontranslated intergenic unannotated ORFs. Black dotted line represents the average for Longest-AUG dataset. c) Average hydrophobicity (Kyte-Doolittle scale) in the last 60 amino acids of CPs, NCPs and iORFs. d) CTTH in noncanonical protein types, in real and shuffled sequences. *0.05 > *P* ≥ 0.01, **0.01 > *P* ≥ 0.001, ***P < 0.0001.

For each sequence, we inferred four degradation determinants: (i) the combined hydrophobicity of the last 30 amino acids of each protein sequence, or C-terminal tail hydrophobicity scores (hereafter CTTH); (ii) the terminal (N-end and C-end) degrons; (iii) the terminal intrinsic disorder regions, or intrinsically disordered regions (IDRs); and (iv) the microautophagy-associated KFERQ-like motifs (Table 1; Fig. 1a). Hydrophobic residues exposed on the surface of misfolded proteins are a known trigger of proteasomal degradation (Fredrickson et al. 2011). By applying CRISPR screens to thousands of human proteins and a large library of random polypeptides, Kesner and colleagues have recently determined that elevated hydrophobicity at the C-terminal tail (CTTH) induce proteasomal degradation (Kesner et al. 2023). Terminal degrons are formed by individual amino acids or short linear motifs placed at either N- or C-terminal of a protein, known as N-end and C-end degrons, which are associated to an increase in degradation propensity (Bachmair et al. 1986; Varshavsky 2019). A high number of degrons have been experimentally verified in humans and other organisms (Kim et al. 2018; Koren et al. 2018; Timms et al. 2019; Timms and Koren 2020; Makaros et al. 2023). The effects on protein stability of some end degrons appear to be conserved across eukaryotes; for instance, glycine acts as a C-end degron in human proteins and is depleted from the last position in proteins across eukaryotes (Koren et al. 2018). Terminal IDRs of 30 or more amino acids have been experimentally shown to correlate to protein degradation both in mammals and budding yeast (van der Lee et al. 2014). In mammals, specific amino acid motifs are also associated with selective protein degradation mediated by the lysosome via chaperone-mediated autophagy (CMA) and endosomal microautophagy (eMI) (Schnebert et al. 2022). Both these processes involve the recognition of KFERQ-like penta-amino acidic motifs by the chaperone heat shock cognate 71 kDa protein (HSC70). KFERQ-like motifs are either canonical (standard) or formed through posttranslational phosphorylation or acetylation (Kirchner et al. 2019).

Neutral expectations for each protein were generated by within-sequence amino acid shuffling that preserves sequence length and residue composition. This permutation approach is standard for protein motif enrichment and provides a conservative baseline for testing composition- and position-dependent features (Davey et al. 2011; Prieto et al. 2014). The same approach has been used across studies of noncanonical protein stability (Kesner et al. 2023; Yang et al. 2024).

### C-terminal Tail Hydrophobicity

An elevated average hydrophobicity in the last 30 amino acids of protein sequence is strongly associated with protein degradation via the proteasome-ubiquitin system (Kesner et al. 2023) (Fig. 1a). Here, we calculated the C-terminal tail hydrophobicity (CTTH) score, across all CPs and NCPs. CTTH scores were obtained using the Kyte-Doolittle scale (Kyte and Doolittle 1982), in which hydrophobicity corresponds to positive values. Although CTTH was on average negative (hydrophilic) in all datasets, we observed significant variation in the overall hydropathy. The MANE isoforms showed the lowest average CTTH (−0.53), indicating diminished hydrophobicity, compared to other canonical isoforms (Wilcoxon rank sum test with FDR correction, *P*  *=* 5.0 × 10^−11^ in all tests, Fig. 1b, Table S3). Moreover, the CTTH score was on average lower and less variable in canonical isoforms starting with a methionine, or AUG isoforms, compared to non-AUG isoforms (Fig. 1b).

Across NCP datasets, CTTH was significantly higher than the MANE isoforms, ranging between −0.36 and −0.41 (*P* < 9.4 × 10^−8^ in all tests, Fig. 1b, Table S3). The mean CTTH difference between MANE isoforms and NCP datasets ranged from 0.11 to 0.17. For the combined NCP dataset, this difference corresponds to a rank-biserial correlation (*r*) of 0.11, indicating a meaningful effect rather than a difference detectable solely because of the large sample size (Table S3). Notably, CTTH was comparable between most NCP datasets and non-AUG canonical isoforms. Putative proteins encoded by non-translated intergenic ORFs (iORF proteins) showed the highest CTTH score. Overall, protein size explained little to no variance in CTTH scores—all datasets had absolute Spearman's correlations <0.085—suggesting that the length of NCPs and CPs does not influence C-end hydrophobicity (Table S4). Functional microproteins shared an elevated CTTH, probably explained by the high frequency of proteins with a C-terminal hydrophobic transmembrane membrane in this dataset (Kesner et al. 2023).

We further observed a marked decrease in hydrophobicity along the C-terminal of the longest canonical isoforms, whereas other CPs, NCPs, and iORF proteins showed no change in hydrophobicity (Fig. 1c). While these results largely align with those of Kesner et al. (2023), they also point to a yet undescribed difference in the C-terminal hydrophobicity between types of canonical proteins (Fig. S1).

Confirming selective constraints toward a decreased C-end hydrophobicity, we found significantly higher CTTH scores in shuffled than original in the MANE isoforms and in the other AUG isoforms (*P* = 1.5 × 10^−188^ and *P* = 6.9 × 10^−50^ ; Fig. 1, Table S3). Conversely, non-AUG isoforms showed comparable CTTH in original and shuffled sequences, which suggests that many of these isoforms might not be translated or have a short half-life. In most NCP datasets, the original sequences shared significantly lower CTTH than shuffled sequences (Fig. 1b, Table S3). Nevertheless, the CTTH gap between original and shuffled sequences was much more pronounced in the MANE dataset than in NCPs. Altogether, these results can be explained by selective constraints acting upon a subset of NCPs to reduce CTTH and prevent degradation.

Across all NCP datasets, CTTH was lower in proteins encoded by uORFs, pseudogenes and internal ORFs (intORFs) than lncRNAs ORFs, although this difference was not statistically significant (Fig. 1d, Table S5). Conversely, downstream ORFs (dORFs) showed significantly higher CTTH scores than other classes of NCPs (Table S5). Differences in degradation propensity among ORF classes may also be affected by specific selective pressures to maintain protein-coding regions, functional RNA structures, and regulatory elements, which may alter the frequency of some degradation determinants. Although CTTH scores were lower in original than shuffled proteins in most cases across datasets, this difference was consistently significant only for uORFs (Fig. 1d, Table S5).

### N-end and C-End Degrons

**Fig. 2.**
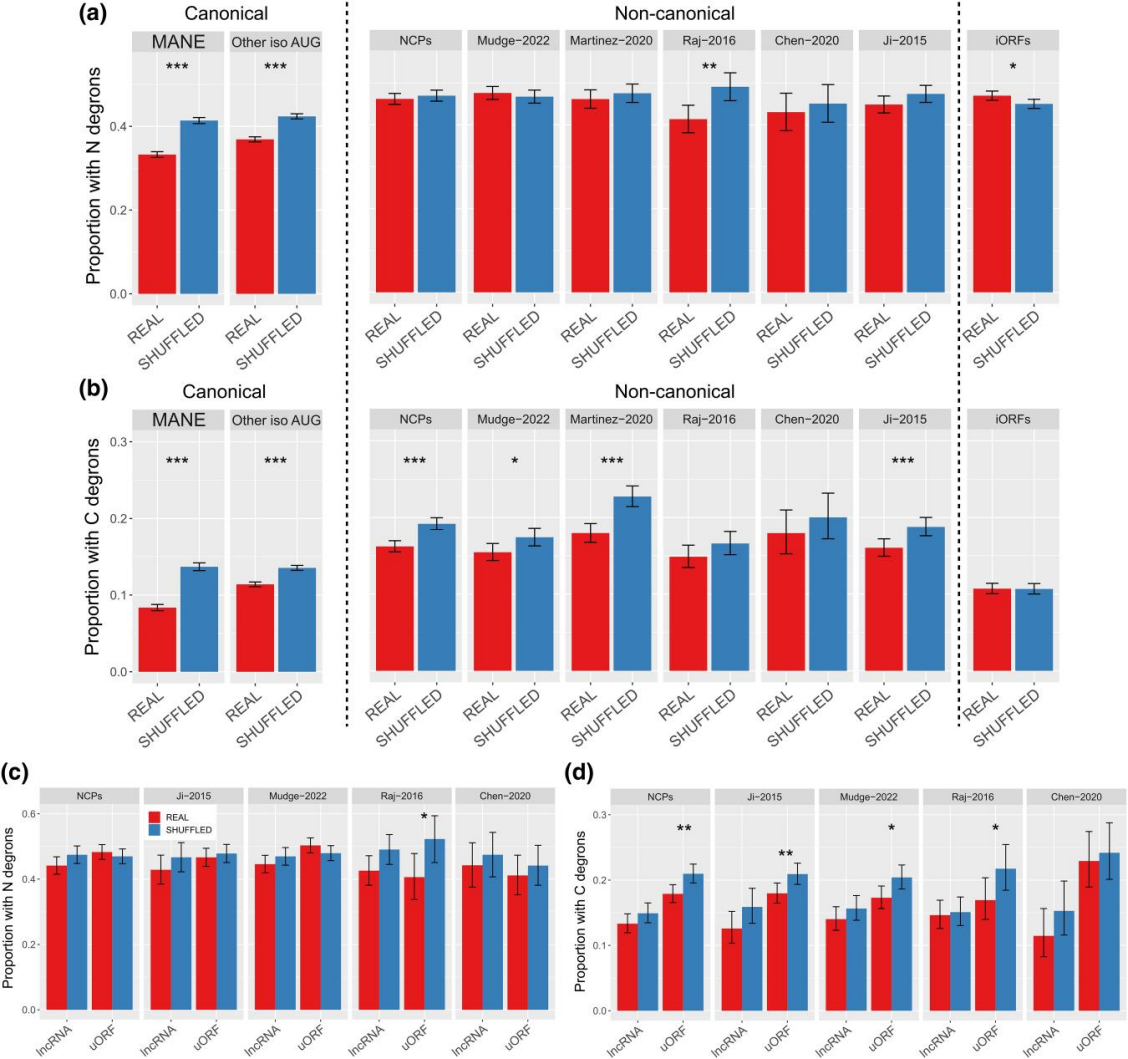
a) Prevalence of 405 N-end degrons in original and shuffled sequences. b) Prevalence of 46 C-end degrons in original and shuffled sequences. c) Prevalence of 46 C-end degrons in peptides encoded by lncRNAs and uORFs in four different datasets. d) Prevalence of 405 N-end degrons in peptides encoded by lncRNAs and uORFs in four different datasets. *0.05 > *P* ≥ 0.01, **0.01 > *P* ≥ 0.001, ***P < 0.0001.

We searched CPs and NCPs for 405 N-end and 46 C-end previously characterized degrons that decrease protein stability in human (Koren et al. 2018; Timms et al. 2019; Timms and Koren 2020) (see Methods). N-end degrons were found in 41% to 48% of NCPs compared to 33% of MANE isoforms (*P* < 3.2 × 10^−5^ between MANE and all NCP datasets, Fig. 2a). Such degrons were also significantly depleted in original compared to shuffled sequences in the two AUG isoform sets and in 94 functional microproteins (*P* < 5.6 × 10^−7^), whereas no significant difference was found in NCP datasets with one exception (Table S6). Similarly, C-end degrons were found in only 8.4% MANE isoforms compared to 11% to 14% other CP isoforms and 15% to 18% of NCPs (*P* < 4.2 × 10^−9^ in all tests, Table S7). Both AUG canonical isoforms and most NCP datasets shared fewer C-end degrons than expected given their shuffled sequences, particularly in MANE sequences (Fig. 2b). Overall, more shuffled sequences consistently contained either type of end degron than original sequences, except for putative proteins encoded by iORFs. Although this discrepancy was not always statistically significant, it is suggestive of a robust selective constraint against the presence of end degrons, particularly in AUG canonical isoforms, and to a substantial degree for C-end degrons in NCPs. Additionally, the increase in both N- and C-end degron frequency corresponds to odds ratios of 1.7 to 2.3 for NCPs relative to MANE proteins (Tables S6 and S7). These values reflect moderate effect sizes consistent across datasets and robust to shuffling controls. Functional microproteins contained comparable number of end-degrons than NCPs, suggesting lower stability than canonical AUG isoforms. Interestingly, putative proteins encoded by intergenic ORFs showed more N-degrons than NCPs but had the second lowest proportion of C-end degrons. This might be the result of specific nucleotide biases in these ORFs.

Across NCP datasets, we observed a significant enrichment of C-end degrons in proteins encoded by uORFs compared to lncRNAs for the Chen-2020 and Ji-2015 datasets (*P* = 0.0018 and *P* = 0.006, respectively; Fig. 2c) and when considering all datasets together (*P* = 0.0006). Conversely, we found no clear trend for N-end degrons among NCP types (Fig. 2d, Tables S8 and S9).

### Terminal Intrinsic Disorder

**Fig. 3.**
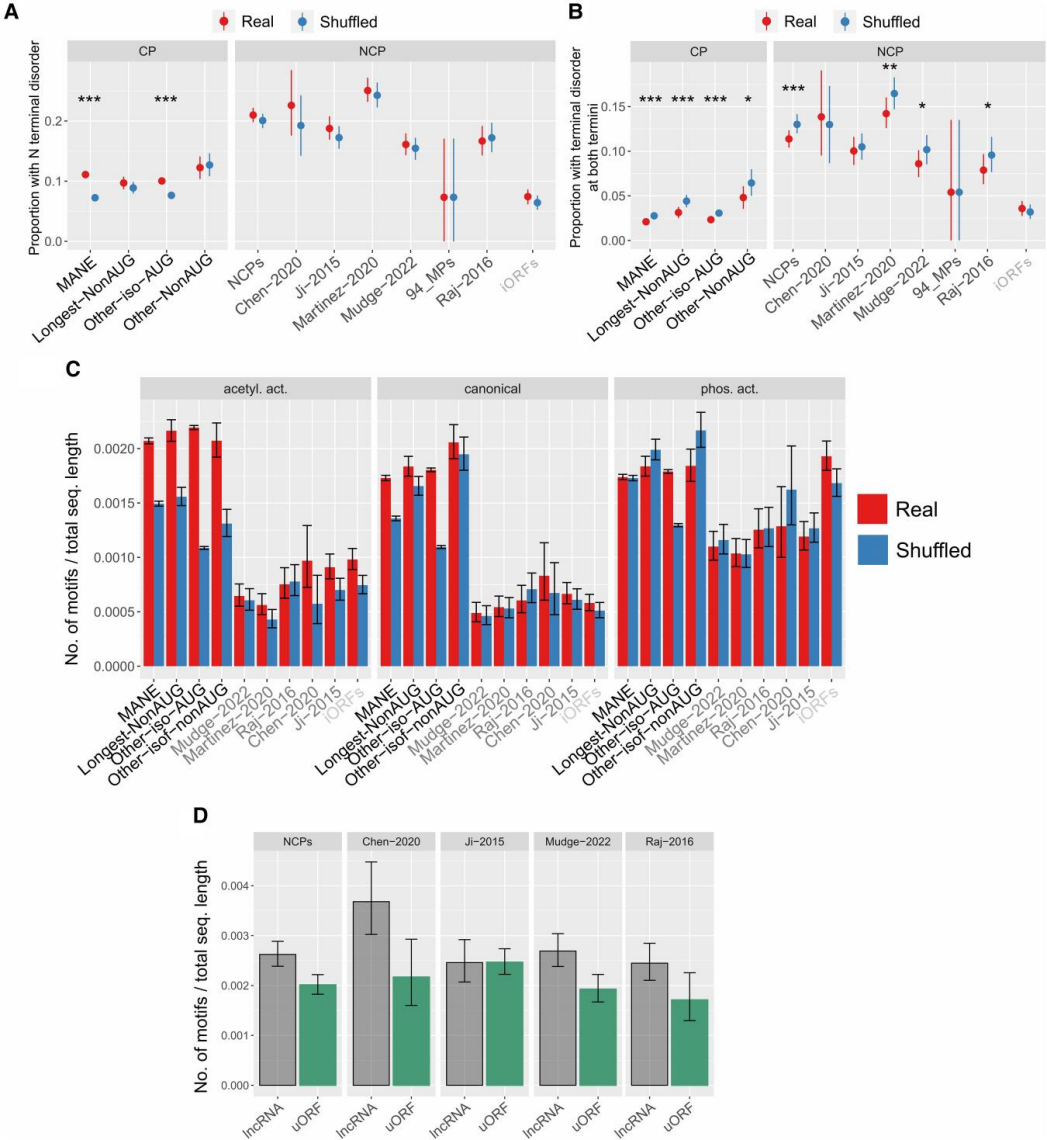
a) Proportion of sequences with N-terminal intrinsically disordered regions. b) Proportion of sequences with C-terminal intrinsically disordered regions. c) Number of KFERQ-like motifs by the total length of each dataset. d) Number of KFERQ-like motifs by the total length of each NCP type dataset. *0.05 > *P* ≥ 0.01, **0.01 > *P* ≥ 0.001, ***P < 0.0001.

We explored if NCPs exhibited different frequencies of IDRs compared to canonical proteins. Only ∼11% of MANE isoforms possessed either a N-terminal or a C-terminal IDRs, as opposed to 14% to 23% of most NCP datasets (Fig. 3a, Fig. S2, Table S10). Intriguingly, fewer MANE shuffled proteins contained terminal IDRs (*P* = 9.8 × 10^−47^ for N-IDRs, *P* = 1.9 × 10^−38^ for C-IDRs), whereas no difference was found across NCP datasets (Table S10). However, the presence of long IDRs at both termini in the same sequence was significantly lower in MANE isoforms with respect to NCPs (∼2% vs. 8% to 14%, *P* < 6.0 × 10^−16^, Fig. 3b). In all CP, combined NCP and in three individual NCP datasets, IDRs at both termini were significantly less frequent in original than shuffled sequences, suggesting a strong constraint against this pattern (Table S10). Interestingly, both functional microproteins and iORF-encoded polypeptides shared some of the lowest frequencies of terminal IDRs. The enrichment of terminal IDRs in NCPs compared to MANE isoforms corresponds to risk ratios of 1.35 to 2.05 and odds ratios of 1.4 to 2.2 (Table S10).

### Microautophagy-Associated KFERQ-Like Motifs

We assessed the possible role of microautophagy in noncanonical protein degradation by inferring KFERQ-like motifs occurrence in protein datasets (Kirchner et al. 2019). As expected, protein length had a strong correlation with the presence of KFERQ-like motifs. Overall, 50% to 78% CPs contained at least one motif compared to 11% to 14% of NCPs and 14% in iORF-encoded putative peptides (Table S11). Moreover, within each dataset, proteins with no KFERQ-like motifs were much shorter than those with motifs (Table S11). After normalizing for protein length, the frequency of KFERQ-like motifs remained consistently higher in CPs than NCPs across the three types of motifs (canonical, phosphorylation-dependent and acetylation-dependent) and significantly higher in CPs than NCPs overall (*P* < 1.3 × 10^−242^ in all tests, Fig. 3c, Table S11). However, KFERQ-like motifs were much more abundant in NCPs than CPs when considering only proteins containing at least one motif (Table S11).

For canonical and acetylation-dependent KFERQ-like motifs, original CPs of all categories were significantly enriched compared to shuffled sequences (*P* < 10^−6^ in all tests), whereas this was consistently not the case for NCPs except one dataset (*P* > 0.05 in all tests, Fig. 3c). Intriguingly, the enrichment in the original sequences was entirely absent for the phosphorylation-dependent motifs. Overall, these findings underlie the importance of autophagy-related pathways in the proteostasis of canonical proteins and of a minority of NCPs. We observed a similar pattern across different types of NCPs, with proteins encoded by lncRNAs sharing higher motif density than those encoded by uORFs in most datasets (*P* < 0.05, Fig. 3d, Table S12). Length-normalized motif counts also showed moderate effect sizes, indicating that this enrichment is not driven solely by large sample sizes (Tables S11 and S12).

### Degradations Determinants and Noncanonical Protein Evolutionary Age

We next tested if degradation propensity in NCPs tend to decrease through time, as observed for CTTH in CPs both in human and mouse (Kesner et al. 2023 ). We found no change in both CTTH and terminal IDRs with age in two NCP datasets, including in NCPs more likely to be functional (Figs. S3a–d, Tables S13 and S14). Similarly, different NCP types showed no change in degradation propensity in relation with their age (Fig. S3e, Table S14). The absence of an evolutionary trend across multiple degradation determinants might simply reflect a biased annotation of increasingly older NCPs as canonical proteins. However, we observed limited to no variation of KFERQ-like motifs with age in CPs as well, suggesting a complex association of gene age with degradation determinants among canonical proteins (Fig. S3f).

### Noncanonical Proteins Stability and Functionality

We next investigated if noncanonical proteins that are more likely to be functional show patterns of stability that align more closely with those observed in canonical proteins. To assess this possibility, we first compared 753 noncanonical sequences with and without an observed phenotype in iPSC and K562 cell lines reported by Chen et al. (2020). We statistically tested phenotypic results from the K562 cell line only and from data obtained in both cell lines, because only a few NCPs were expressed in the iPSC cell line only (Table S15). CTTH scores were significantly lower in NCPs with a reported phenotype in K562 and in both cell lines compared to those with no phenotype (*P* = 0.037 and *P* = 0.012, respectively), whereas no significant trend was found for degrons and IDRs (Fig. S4, Table S15). The reduction in CTTH among NCPs with cellular phenotypes corresponds to Cohen's *d* = 0.28 to 0.34 (Table S15), supporting a biological difference between the two datasets. NCPs with a phenotypic effect encoded by lncRNAs and uORFs also shared lower, but not statistically significant, CTTH values than NCPs with no phenotype. Original sequences shared lower CTTH than shuffled sequences in proteins with phenotypic effect, but this was significant only for data reported in the K562 line (Table S15). Interestingly, KFERQ-like motifs were consistently less frequent in NCPs with a phenotype (Poisson regression, *P* < 0.001), and in original versus control sequences (*P* > 0.5), suggesting depletion of CMA motifs in these potentially functional proteins (Table S15).

Second, we analyzed NCPs from a catalog of Ribo-seq ORFs that have been found in one (“weak”: 4,179 NCPs) or more than one (“robust”: 3,085 NCPs) studies (Mudge et al. 2022). CTTH was significantly lower in the robust dataset (pairwise logistic regression, *P* = 0.012), whereas all IDRs were significantly higher in the robust dataset (pairwise logistic regression, *P* < 0.05). N-end degrons, C-end degrons, and KFERQ-like motifs occurred more often in the robust datasets but not significantly so (Table S16; Fig. S5). However, both CTTH values and the frequency of C-end degron were lower in original than shuffled sequences of the robust dataset only, indicating possible selection toward increased stability in these NCPs. The potential role of CTTH variation between robust and weak NCPs is supported by moderate Cohen's *d* (Table S16).

### Degradation Signatures and LncRNA Localization

LncRNAs occurring only in the nucleus cannot associate to ribosomes and should thus encode putative proteins that freely accumulate degradation determinants compared to lncRNAs primarily found in the cytoplasm. To test this hypothesis, we analyzed data from the LncATLAS database, which contains information on the subcellular localization of lncRNAs in human expressed as Relative Concentration Index, or RCI, between two compartments (Mas-Ponte et al. 2017) (see Methods). We identified 3,379 lncRNAs with both nucleus/cytoplasm RCI expression ratio and Ribo-seq data. As expected, ribosome-associated lncRNAs were significantly more likely to localize in the cytoplasm compared to non-translated lncRNAs (Fisher's exact test, *P* = 0.0001, Table S17). Among lncRNAs with evidence of protein synthesis, those localized in the cytoplasm encoded proteins with significantly lower CTTH than proteins encoded by nucleus-located lncRNA (Fisher's exact test, *P* = 0.0089, Table S17). No other degradation determinants showed significant difference among proteins encoded by cytoplasmic and nuclear lncRNAs, possible due to the undetected synthesis of functional proteins from lncRNAs that reportedly localize in the nucleus but occur in the cytoplasm of cell types not reported in the LncATLAS database.

A recently reported lncRNA subcellular localization dataset from human H9 embryonic stem cells (Guo et al. 2020) was also analyzed applying a cytoplasmic ratio threshold of 0.5 to identify lncRNAs the preferentially localize in the cytoplasm (see Methods). As expected, the cytoplasmic ratio was higher in 1,601 lncRNAs with Ribo-seq detection than 2,102 lncRNAs without a match to Ribo-seq data (Fischer's exact test, *P* = 0, Table S18). Additionally, the CTTH value was significantly lower in peptides encoded by lncRNAs with cytoplasmic ratio >0.5 and thus more likely to be associated to ribosomes (*P* = 0.0064, Table S18).

### C-terminal Tail Hydrophobicity and Noncanonical Tail-Anchored Proteins

Tail-anchored (TA) proteins are characterized by a C-terminal transmembrane domain (TMD) and thus present high level of C-end hydrophobicity (Costello et al. 2017; Guna and Hegde 2018). In mammals, TA proteins are recognized by the SGTA/BAG6 checkpoint before being delivered to other mediators of their transmembrane insertion (Mock et al. 2015; Costello et al. 2017), preventing proteasomal degradation despite elevated CTTH (Kesner et al. 2023). Intriguingly, most known human functional NCPs are associated to cellular membranes, suggesting a pathway to functional recruitment of NCPs as novel TA proteins (Kesner et al. 2023). We tested this hypothesis more broadly by assessing if high CTTH scores in NCPs are due to an enrichment for TA proteins among noncanonical polypeptides. TA-proteins were predicted across all datasets as described in Methods. A total of 513 TA-proteins with no redundant C-terminal, out the 573 TA-proteins previously reported in human (Fry et al. 2021), were also analyzed.

**Fig. 4.**
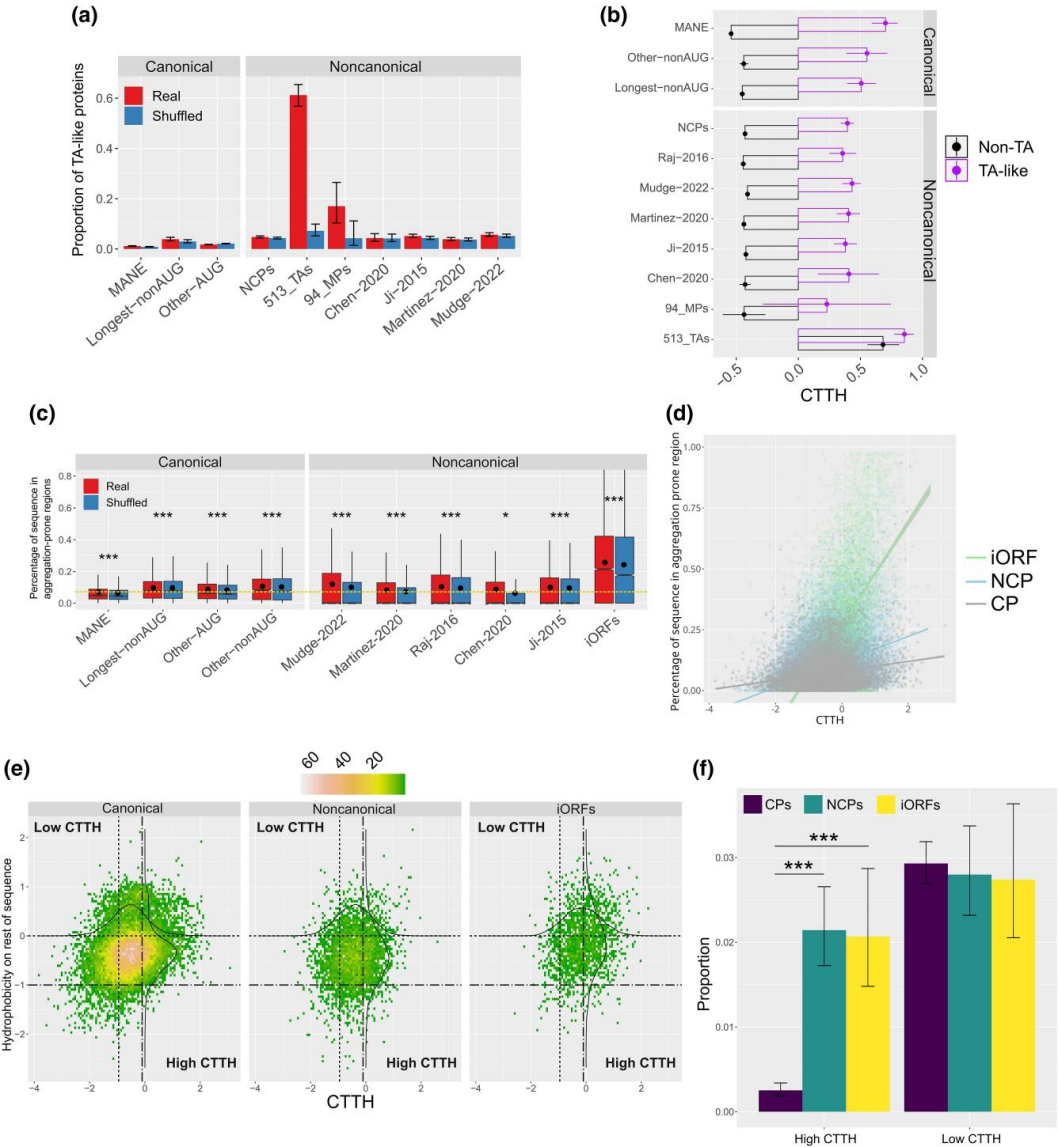
a) Frequency of predicted TA-like proteins in AUG and non-AUG CPs, NCPs and 59 functional NCPs. b) CTTH in TA and non-TA proteins in AUG and non-AUG CPs, NCPs, 59 functional NCPs and 513 human TA proteins. c) Aggregation levels in canonical and noncanonical protein datasets. In all datasets, original sequences showed significantly higher aggregation than shuffled sequences. d) CTTH-Aggregation correlation. Red squares: NCPs. Blue circles: CPs. Black triangles: proteins encoded by intergenic ORFs. e) Distributions of CTTH hydrophobicity in the rest of the protein. Applied cut-offs are drawn as dotted lines. f) Proportion of CPs and NCPs with high and low CTTH values compared to the rest of the protein. *0.05 > *P* ≥ 0.01, **0.01 > *P* ≥ 0.001, ***P < 0.0001.

The proportion of NCPs with TA protein-like features was significantly higher compared to MANE proteins (4.8% vs. 1.1%; Fisher's exact test, *P* < 0.0001), particularly among 94 functional microprotein isoforms—83 of which are non-redundant at the C-end and were thus analyzed—reported by Kesner et al. (2023) (Fig. 4a, Table S19). However, only ∼4% to 6% of sequences across NCP datasets had predicted TA structures. To determine if NCPs with tail-anchored protein features account for the elevated average CTTH across all NCPs, we analyzed CTTH values after removing predicted TA sequences. CTTH scores remained significantly higher in all but one NCP dataset compared to MANE isoforms, indicating that TA-like features are not the main driver of higher CTTH in NCPs (*P* < 5.5 × 10^−6^, Fig. 4b, Table S19).

### Degradation Determinants and Protein Aggregation

The formation of toxic aggregates from misfolded proteins is a major cause of cell stress and is associated with a range of diseases (Soto and Pritzkow 2018). Because selection acts to reduce proteins propensity to assemble into large fibrillar structures (Monsellier and Chiti 2007), we hypothesized that degradation determinants could correlate with aggregation propensity. Similar to KFERQ-like motifs, the presence of aggregation-prone amino acids is correlated with protein length. Accordingly, most CPs showed at least some level of aggregation propensity, which was instead observed in less than 50% of NCPs. Interestingly, more than 66% of putative proteins from iORFs contained aggregation residues, suggesting that spurious translation of intergenic ORFs might be deleterious and that NCPs represent a subset of potentially less harmful sequences than iORFs. When normalizing for sequence length, we found that only 5% to 7% of CP sequences contained aggregation-associated amino acids compared to 8% to 1% of NCPs (*P* < 3.4 × 10^−6^) and 24% in putative proteins encoded by intergenic ORFs (Fig. 4c; Table S20). There was a significantly higher aggregation propensity in original than shuffled sequences in all data sets, supporting the view that most aggregation-prone amino acids are required for protein function and that cellular mechanisms prevent homodimers precipitation in physiological conditions.

CTTH was higher in proteins with aggregation propensity than those without, confirming our predicted association of degradation propensity with aggregation. We also noticed that NCPs exhibited a stronger CTTH-aggregation correlation than CPs (Fig. 4d). However, this correlation increased in shuffled CPs, while it does not change between original and shuffled NCPs (Table S21). Thus, the higher CTTH-aggregation correlation in NCPs might be due to amino acid composition bias rather than selection pressure to decrease stability in more aggregation-prone NCPs. Among other degradation determinants, N-end and C-end degrons showed a weak correlation with aggregation propensity, whereas IDRs were significantly depleted in aggregation-prone proteins, as expected given the inverse correlation between disordered regions and aggregation (Tables S20 and S21).

### Patterns of CTTH Enrichment and Depletion and Noncanonical Protein Function

Stable proteins maintain low C-end hydrophobicity regardless of the overall hydropathy of the whole protein (Kesner et al. 2023). Accordingly, the presence of a combined low CTTH and elevated hydrophobicity in the rest of the sequence is indicative of selective pressure toward high stability. We examined this pattern in noncanonical proteins and identified 111/3,966 sequences with highly hydrophilic C-end and a hydrophobic rest of the sequence (see Methods, Fig. 4e and f, Table S22). This is a statistically equivalent proportion than in canonical proteins (534/18,277; Fisher's exact test, *P* = 0.72) and in putative proteins encoded by iORFs (49/1,789; Fisher's exact test, *P* = 0.93).

We also explored the opposite scenario, whereby natural selection would favor the accumulation of hydrophobic residues at the C-end in a generally non-hydrophobic protein, possibly to fine-tune the activity of short-living regulatory proteins. We discovered 85 NCPs with both a high CTTH and a strongly hydrophilic rest of the protein, compared to only 76 CPs with the same pattern of hydropathy (Fig. 4e and f, Fisher's exact test*, P* < 0.00001, Table S22). Similarly, we found an excess of high CTTH in hydrophilic putative proteins encoded by iORFs (37/1,789, Fisher's exact test, *P* < 0.00001), whereas there was no significant difference between iORF proteins and NCPs (*P* = 0.92). Altogether, these results suggest that NCPs retain a similar hydropathy profile along their sequence compared to non-expressed iORF proteins and point out an intrinsic propensity of a fraction of ncORFs to encode degradation-prone proteins.

## Discussion

The biological impact of noncanonical proteins (NCPs) is controversial due to their potential instability, among other reasons. Here, we tested if NCPs showed lower average stability compared to canonical proteins by assessing the frequency of degradation determinants signatures across tens of thousands of human proteins. We investigated three determinants associated with proteasomal degradation: C-terminal tail hydrophobicity, or CTTH (Kesner et al. 2023), N- and C-end degrons (Varshavsky 2019; Timms and Koren 2020), and terminal intrinsically disordered regions (van der Lee et al. 2014). We also examined the occurrence of amino acid motifs necessary for autophagy-mediated degradation (Kirchner et al. 2019; Schnebert et al. 2022). NCPs shared a higher frequency of the three proteasomal-related degradation determinant compared to canonical AUG protein isoforms but a lower frequency of KFERQ-like motifs associated with CMA and eMI. The latter finding is in partial disagreement with results from a recent study showing that some NCPs are stabilized when lysosome/autophagy-mediated pathways are inhibited (Yang et al. 2024). This could depend on the limited number of NCPs analyzed experimentally or indicate that lysosomal pathways other than CMA and endosomal microautophagy are involved in NCPs degradation, including non-CMA/eMI microautophagy and macroautophagy pathways (Yim and Mizushima 2020). Several lysosomal selective routes beyond CMA and endosomal microautophagy are known (Lamark and Johansen 2021), including macroautophagy pathways relying on LC3/ATG8-binding via LIR/AIM motifs (Johansen and Lamark 2020) and, for membrane proteins, the endocytic pathway that target proteins to the lysosome using both specific signals (YXXΦ, [DE]XXXL[LI], NPXY) and ubiquitination (Braulke and Bonifacino 2009). However, these pathways lack a single, specific linear motif on soluble cargo akin to the KFERQ-like motif and were not investigated.

Our conclusions are supported both by statistical significance and meaningful effect sizes. Across all degradation determinants, noncanonical proteins differ from canonical isoforms by small-to-moderate quantitative effects. CTTH differences typically yielded rank biserial correlation *r* = 0.1 to 0.3, whereas degron enrichments corresponded to odds ratios of 1.7 to 2.6 and terminal-IDR frequencies were 1.4 to 2.2 times higher in NCPs (Tables S3 and S7–S12). These effects remain after controlling for amino-acid composition by sequence shuffling, suggesting that selection contributes to the observed patterns.

These findings underscore the essential role of multiple surveillance pathways in preventing the expression of potentially harmful proteins, significantly enhancing our understanding of processes regulation translation of ncORFs. Notably, the enrichment of three degradation determinants in NCPs was consistent across ribo-seq datasets analyzed with different tools, including the less performative riboHMM program (Zhang et al. 2017). Additionally, our results strengthen and expand the role of CTTH in regulating translation errors recently reported by Kesner et al. (2023). First, we showed that the main human protein isoforms (MANE dataset) share lower CTTH values compared to both non-AUG canonical proteins and NCPs. MANE isoforms also exhibited the strongest CTTH differences between original and shuffled sequences, a strong indicator of selective constrains, and reduced frequency of N-end degrons and IDRs at both protein termini. Second, our discovery of different CTTH values in cytoplasmic versus nuclear lncRNAs underscores the importance of this signature in discriminating translated from untranslated ORFs in lncRNAs. Third, the comparison of CTTH scores with hydropathy in the rest of the protein unveiled hundreds of proteins with either enriched or depleted C-end hydrophobicity, which could result from selective pressure toward a reduced or increased stability, respectively. Notably, sequences with lower than expected stability were significantly more common among NCPs that CPs.

Noncanonical proteins inferred to be functional are expected to be depleted of degradation features. Accordingly, we observed lower CTTH scores and KFREQ-like motif frequencies in NCPs with a cellular phenotype (Chen et al. 2020). However, we caution that this interpretation will require further experimental confirmations of a phenotypic effect of NCPs identified through CRISPR/Cas9 knock-out experiments. Noncanonical proteins expressed across multiple studies (Mudge et al. 2022) also shared lower CTTH scores and a paucity of some degradation determinants in comparison with NCPs found in a single study. Thus, experimental phenotypic assays might be more effective in identifying potentially functional noncanonical proteins compared to expression consistency across studies. These findings should inform the ongoing annotation efforts of functional human NCPs (Mudge et al. 2022).

Protein degradation determinants must be accessible to mediators of degradation pathways in order to exert their function, a possible limitation in analyses of stability based on proteins' primary sequences. For example, KFERQ-like motifs are effective when exposed on the surface of a protein, thus available to autophagy via binding to the chaperone HSC70 (Kirchner et al. 2019). However, given that C-terminal tail, degrons, and terminal IDRs occur in protein regions that are likely to be exposed (Jacob and Unger 2007), most of our findings appear to be robust to possible biases in access to degradation signals.

Most known human functional NCPs are localized on cellular membranes. We confirmed that the majority of these proteins contain a C-terminal TMD, a signal present in tail-anchored canonical proteins to allow their appropriate routing to cellular membranes via the SGTA/BAG6 checkpoint (Mock et al. 2015). TMD are highly hydrophobic and cause the elevated CTTH of TA proteins and membrane-bound NCPs. Since only a small fraction of all NCPs contains a C-terminal TMD, it is unlikely that many NCPs gain functionality by becoming TA proteins.

Because insoluble proteins forming deposits are deleterious in the cellular environment, we hypothesized that NCPs might accumulate degradation determinants at higher rate than CP due to selection to diminish the impact of aggregates. Although NCPs shared stronger aggregation signatures than CPs, we found no evidence that degradation determinants have higher frequency in NCPs that are more likely to aggregate. Thus, the elevated degradation propensity of NCPs could result from amino acid compositional biases, rather than from selection to decrease their potential cellular toxicity by affecting their stability.

Overall, our study provides an innovative approach to predict degradation propensity in both canonical and noncanonical proteins. We unveiled significant differences in protein stability among >90,000 annotated canonical isoforms that complement and expand on analyses of degradation rates in a few thousands human isoforms (Zecha et al. 2018; Salovska et al. 2020). This could result from different biological roles across isoforms that are associated with variation in their expression pattern, structure or genetic network. The degradation determinant predictions emphasize the need to further characterize the relationships between stability and biological impact of protein isoforms. In this regard, a more accurate annotation of non-AUG isoforms will be critical, including systematic analyses of upstream AUG codons reported in some human gene 5′ UTRs (Loughran et al. 2020). Finally, our finding of a consistently lower stability across noncanonical proteins in comparison to canonical isoforms independently substantiate experimental data that have led to the DRiPs hypothesis, and demonstrate the power of protein degradation signatures in assessing the impact of the “dark proteome” on human biology.

## Methods

### Canonical Protein Sequence Datasets

Canonical protein sequences were obtained by integrating data from the Ensembl v105 database (Harrison et al. 2024) and the Matched Annotation from the NCBI and EMBL-EBI (MANE) database release 1.3 (Morales et al. 2022). The MANE database contains single representative transcripts of each human protein-coding locus selected on the basis of evolutionary conservation and expressed of each protein-coding exon, providing a robust set of primary protein isoforms for ∼99% of all human genes. The 117,909 Ensembl v105 proteins were processed as follows. Protein sequences shorter than 60 amino acids or containing the non-standard amino acid “U” were removed. Additionally, we applied a length threshold of 60 amino acids to allow hydrophobicity level comparisons between the C-terminal tail and the rest of the protein (see also Methods section “*Hydropathy and functional noncanonical proteins*” below). The remaining 91,003 sequences were separated in two groups according to the presence/absence of a standard AUG starting codon in the corresponding transcripts. The AUG group was further divided into a 18,802 set of proteins also reported in the MANE database (after removing 170 sequences that were 100% identical to other longer MANE sequences) and a second set of 67,704 protein sequences, which in our analyses are labeled “MANE” and “Other-isoforms-AUG” datasets, respectively (Tables S1 and S3).

Proteins sharing an identical sequence in the C-terminal 30 amino acids were identified to remove redundancy in the CTTH analyses described below. Non-AUG datasets were further divided into those including only the longest CDS isoforms of each locus and those containing the remaining protein isoforms, labeled “Longest-non-AUG” and “Other-non-AUG” throughout the study, respectively (Tables S1 and S3). This distinction was made assuming a possible different biological impact between the longest protein isoforms and other isoforms encoded by the same locus, in the absence of an equivalent of the MANE database for non-AUG isoforms. We notice that the vast majority of non-AUG isoforms are encoded by loci that also express AUG isoforms.

### Noncanonical Protein Sequence Datasets

We obtained noncanonical protein sequences from four primary analyses and one metadata study. All noncanonical protein (NCP) datasets were obtained from Supplementary Tables of the correspondent articles unless differently specified. In the Raj et al. dataset, 122 mCDS and uaCDS with redundant IDs were removed. Genome coordinates of ORFs annotated in hg19 were converted to hg38 coordinates using liftover for the Chen et al. dataset (Chen et al. 2020) and Raj et al. dataset (Raj et al. 2016) in order to obtain the corresponding coding regions. Fasta sequences of ORFs were extracted from the hg38 genome assembly using gtf files and the bedtools suite (Quinlan and Hall 2010). ORFs were translated into protein sequences using the getorf script in the EMBOSS suite (Rice et al. 2000).

The 15,783 sequences from the five datasets were assembled into a combined set of 11,666 NCPs after removing redundant identical sequences. This catalogue was further screened for overlap with CPs by performing a BLASTP search (Camacho et al. 2009) against all CPs. We applied two thresholds, 90% sequence identity and 90% coverage with CPs, and filtered out 426 NCPs. The BLASTP analysis was set with default parameters except evalue = 1e^−10^. For both CP and NCP datasets, further degradation determinant-specific filtering steps were performed to eliminate redundancy, as described in the section “*Degradation signals analyses*” below.

### Unannotated Intergenic Genomic ORFs

The hg38 human genome assembly was downloaded together with the gtf files of RefSeq genes (table ncbiRefSeq) and lncRNA and TUCP (Transcripts of Uncertain Coding Potential) from the UCSC Genome Browser. The gtf files of the comprehensive GENECODE V43 dataset was obtained from the GENCODE website https://www.gencodegenes.org/human/. A lncRNAs gtf file (LNCipedia v5.2) was downloaded from https://lncipedia.org/download (Volders et al. 2019). Unix command lines were used to extract and combine exon sequences from the gtf files into a single file of transcribed exons, which were then merged with bedtools merge. Coordinates of the human genomic regions complementary to transcribed regions were obtained with bedtools subtract on a bed file of hg38. Fasta DNA sequences of these regions were retrieved using bedtools getfasta (Quinlan and Hall 2010). Sequences containing “N” bases and those <1,000 bp were removed. The coordinates of 1,000 remaining sequences were randomly selected and used to obtain the corresponding genomic DNA from the UCSC Genome Browser. We then used getorf from the Emboss suite (Rice et al. 2000), to translate the 1,000 random genomic regions and obtained 8,136 non-overlapping iORFs applying of minimum 90 bp that were used as negative control in our analyses.

### Degradation Signals Analyses

The average hydrophobicity was calculated using the Kyte and Doolittle scale (Kyte and Doolittle 1982), which show high correlation with degradation rates in human proteins (Kesner et al. 2023), using the GRAVY portal https://www.gravy-calculator.de. For each dataset, we analyzed only sequences longer than 59 amino acids, and redundant sequences with identical 30 amino acids at the C-end were removed prior to the CTTH calculation, keeping the longest isoforms (Table 1).

Degrons have been experimentally ranked by their contribution to protein instability throughout the differential protein stability index, or ΔPSI. This parameter is calculated as the mean difference in protein stability between polypeptides that contain the degron motif at the N terminus after the initiator methionine (or at the C-end) and polypeptides that contain the same motif at any other internal position (Koren et al. 2018; Timms et al. 2019; Timms and Koren 2020). Degrons with a ΔPSI ≤ −0.4 were retained, as this threshold is well below the ΔPSI value of −0.5 that includes destabilizing motifs (Timms et al. 2019). A total of 405 likely functional human N-degrons falling below this threshold were obtained from Timms et al. (2019). Forty-six C-degrons with ΔPSI ≤ −0.4 were obtained from Koren et al. (2018). Analyses were conducted both with the full list of 405 N-degrons and 46 C-degrons as well as with the top 20 motifs in each group according to their ΔPSI. Sequences with identical 30 amino acids at their N-end and C-end were removed from the N-degron and C-degron analyses, respectively. For each dataset, we analyzed N-end degrons and C-end degrons after retaining entries with unique 10 amino acids sequences at the N- and C-end, respectively (Table 1).

IDR data were obtained using IUPred2a (Erdos and Dosztanyi 2020) with the option “long disorder” and by parsing the results with in-house python scripts to identify N-terminal and C-terminal IDRs longer than 30 amino acids. Although both terminal and internal IDRs decrease protein stability, we focused only on terminal IDRs because of the length bias of noncanonical proteins. Only NPCs longer than 59 amino acids were analyzed. Redundant sequences with identical sequences at the N-end and C-end were removed before searching for N-terminal and C-terminal IDRs, respectively, keeping the longest isoforms (Table 1).

KFERQ-like motifs were inferred using the “motifs from sequence” option in the KFERQfinder portal (https://rshine.einsteinmed.edu) with default settings to search for canonical motifs, phosphorylation-activated motifs and acetylation-activated motifs (Kirchner et al. 2019). All sequences in the datasets were included in this analysis (Table 1)

### Protein Age

Proteins from the Mudge et al. dataset (Mudge et al. 2022) were divided by Sandmann et al. (Sandmann et al. 2023) in four evolutionary ages: human-specific, old world monkey (Catarrhini-specific), primatomorpha (shared by primates and colugos), and conserved across mammals. Information for the noncanonical proteins in Vakirlis et al. (2022) included nineteen nodes along the human phylogeny from Vertebrates to human specific. To estimate CTTH from at least four sequences per age group, we collated several age groups. GenTree was used to recover the evolutionary age of human canonical genes (Shao et al. 2019).

### Known Functional Microproteins

A list of 64 known functionally characterized microprotein genes was obtained from Kesner et al. (2023). Microprotein sequences were retrieved from original papers and the NCBI Protein database. We found some redundancy in the original list, with some pairs of identical sequences known under different names, ie mitoregulin and MPM, BNLN and the TUNAR isoform 2, and myoregulin and MRLN. A total of 94 unique isoform sequences were retained in the final dataset. Cellular phenotypic data from CRISPR assays were obtained from Chen et al. (Chen et al. 2020).

### LncRNA Subcellular Localization

Subcellular localization of 6,768 human GENCODE annotated lncRNAs were downloaded from LncATLAS (https://lncatlas.crg.eu). Raw data (2023-05-25_lncATLAS_all_data.csv) were parsed to retain only noncoding sequences. In LncATLAS, the prevalence of a given lncRNA for a subcellular compartment is reported as the Relative Concentration Index, or RCI, corresponding to the log_2_ transformed ratio of the RPKM values from two compartments.

Rows with “ratio2” values, corresponding to the “relative concentration index” (RCI) or log2-transformed ratio of FPKM between the cytoplasm and the nucleus, were retained. When multiple ratio2 values for the same lncRNA were averaged. To retrieve CTTH values for these lncRNAs, we performed Blast analyses against all Ribosomal profiling datasets from Table S1 with calculated CTTH. cDNA sequences of the 6,768 GENCODE lncRNAs were obtained from Ensembl and used in a tBlastn with the following parameters: -ungapped -comp_based_stats F -seg yes -evalue 1e-03 -max_hsps 20 -max_target_seqs 20. Only Blast hits with 100% coverage and identity with at least one RiboSeq sequence were retained. After removing LncATLAS entries with multiple hits, we obtained 3,379 lncRNAs with assigned CTTH values.

Subcellular localization data (nucleus vs. cytoplasm) for lncRNAs in human H9 embryonic stem cells were obtained from Guo et al. (2020). The lncRNAs localization was calculated using the cytoplasmic ratio, corresponding to the cytoplasmic FPKMs divided by the sum of cytoplasmic and nuclear FPKMs. We mapped genome coordinates of 4,804 lncRNAs with subcellular information onto the GENCODE v28lift37 lncRNA dataset and found 3,706 unequivocally assigned lncRNA transcripts. tBlastn analyses of these sequences were performed against all Ribosomal profiling datasets from Table S1 with calculated CTTH using the following parameters: -ungapped -comp_based_stats F -seg yes -evalue 1e-03 -max_hsps 20 -max_target_seqs 20. Only Blast hits with 100% coverage and identity with at least one RiboSeq sequence were retained. After removing entries with multiple hits, we obtained 1,601 lncRNAs with assigned CTTH values.

### Prediction of Transmembrane Domains and Tail-anchored (TA) Proteins

TMDs and signal peptides were predicted using the TOPCONS web server using the remote batch script topcons2_wsdl.py (Tsirigos et al. 2015). The results were parsed using in-house python scripts. We implemented a modified version of the pipeline described by Fry et al. (2021) and used the following criteria to define tail-anchored TMDs: absence of a signal peptide and of multiple TMDs, presence of a single TMD no longer than 29 amino acids, a cut-off length for the C-end tail after the TMD of 29 amino acids. Non-TA proteins with TMDs were identified as any remaining sequence with a TMD. Our approach was robust to false positives, as we identified ∼60% of TA proteins described by Fry et al. (2021), likely due to the elevated sensitivity to signal peptide detection in TOPCONS, which are considered incompatible with TA proteins. Our datasets also included 83 isoforms from a list of 59 non-redundant functionally characterized microproteins reported in Kesner et al. (2023), most of which have been found to localize on cellular membranes and thus are expected to possess TMDs.

### Protein Aggregation

Protein aggregation was predicted with PASTA v2.0 (Walsh et al. 2014) in “self” mode, allowing to estimate aggregation propensity of homodimers. Percentage of residues likely to aggregate was obtained by dividing the number of residues with free energy below −5 and probability higher than 0.01 by the length of the protein sequence. The free energy is calculated by a function estimating the stability of beta structures that pair between stretches of protein sequences. Because the beta-pairings vary between sequences with the same amino acidic composition, different overall aggregation estimates were obtained for original and shuffled sequences.

### Hydropathy and Functional Noncanonical Proteins

We calculated the Kyte-Doolittle index for the last 30 amino acids, ie the CTTH score, and for the rest of the sequence in noncanonical proteins longer than 59 amino acids. Proteins with CTTH below the lowest quartile in AUG canonical longest isoforms (CTTH < −0.963) and a hydrophobic (positive) Kyte-Doolittle index in the rest of their sequence were retrieved as possible examples of proteins selected to reduce degradation propensity. To identify cases that represented the opposite pattern of selection to increase instability, we used as thresholds a CTTH above the highest quartile of AUG canonical proteins of −0.110 (after removing proteins with a TMD at the C-end as predicted using TOPCONS (Tsirigos et al. 2015)) and a highly hydrophilic rest of the protein (Kyte-Doolittle index < −1).

### Statistical Analyses

All statistical analyses and data representations were performed using the R environment (v4.3) (R Core Team 2021). For comparisons of continuous or ordinal data (eg CTTH scores), pairwise comparisons between datasets were performed using the Wilcoxon rank-sum test. Paired comparisons within each dataset (original vs. shuffled; red text on diagonal) were conducted using the Wilcoxon signed-rank test. For these analyses, the effect size *r* (rank-biserial correlation) was calculated using the rstatix package, and *P*-values were adjusted for multiple comparisons using the Benjamini–Hochberg (BH) method. For the analyses of binary data (eg presence of degrons in the N-terminal), multiple pairwise comparisons between datasets (white background, black text) were performed using Fisher's exact test, while paired comparisons (original vs. shuffled; red text) were conducted using McNemar's test. For these tests, the odds ratio was calculated as the measure of effect size, and *P*-values were similarly corrected using the BH method. Confidence intervals of proportions were calculated with the R function prop.test. All *P*-values and their corresponding effect sizes are described in the Supplementary Tables, except where otherwise indicated in the main text.

## Supplementary Material

evag009_Supplementary_Data

## Data Availability

All protein fasta sequences and results generated in this study are deposited in the Figshare repository available at https://doi.org/10.6084/m9.figshare.25639029.v2.

## References

[evag009-B1] Abascal F, et al Loose ends: almost one in five human genes still have unresolved coding status. Nucleic Acids Res. 2018:46:7070–7084. 10.1093/nar/gky587. 29982784 PMC6101605

[evag009-B2] Anderson DM, et al A micropeptide encoded by a putative long noncoding RNA regulates muscle performance. Cell. 2015:160:595–606. 10.1016/j.cell.2015.01.009. 25640239 PMC4356254

[evag009-B3] Azam S, Yang F, Wu X. Finding functional microproteins. Trends Genet. 2025:41:107–118. 10.1016/j.tig.2024.12.001. 39753408 PMC11794006

[evag009-B4] Bachmair A, Finley D, Varshavsky A. In vivo half-life of a protein is a function of its amino-terminal residue. Science. 1986:234:179–186. 10.1126/science.3018930. 3018930

[evag009-B5] Barbosa C, Peixeiro I, Romão L. Gene expression regulation by upstream open Reading frames and human disease. PLoS Genet. 2013:9:e1003529. 10.1371/journal.pgen.1003529. 23950723 PMC3738444

[evag009-B6] Basrai MA, Hieter P, Boeke JD. Small open reading frames: beautiful needles in the haystack. Genome Res. 1997:7:768–771. 10.1101/gr.7.8.768. 9267801

[evag009-B7] Braulke T, Bonifacino JS. Sorting of lysosomal proteins. Biochim Biophys Acta. 2009:1793:605–614. 10.1016/j.bbamcr.2008.10.016. 19046998

[evag009-B8] Brunet MA, et al OpenProt 2021: deeper functional annotation of the coding potential of eukaryotic genomes. Nucleic Acids Res. 2021:49:D380–D388. 10.1093/nar/gkaa1036. 33179748 PMC7779043

[evag009-B9] Brunet MA, Levesque SA, Hunting DJ, Cohen AA, Roucou X. Recognition of the polycistronic nature of human genes is critical to understanding the genotype-phenotype relationship. Genome Res. 2018:28:609–624. 10.1101/gr.230938.117. 29626081 PMC5932603

[evag009-B10] Calvo SE, Pagliarini DJ, Mootha VK. Upstream open reading frames cause widespread reduction of protein expression and are polymorphic among humans. Proc Natl Acad Sci U S A. 2009:106:7507–7512. 10.1073/pnas.0810916106. 19372376 PMC2669787

[evag009-B11] Camacho C, et al BLAST+: architecture and applications. BMC Bioinformatics. 2009:10:421. 10.1186/1471-2105-10-421. 20003500 PMC2803857

[evag009-B12] Chen J, et al Pervasive functional translation of noncanonical human open reading frames. Science. 2020:367:1140–1146. 10.1126/science.aay0262. 32139545 PMC7289059

[evag009-B13] Chong CE, et al Integrated proteogenomic deep sequencing and analytics accurately identify non-canonical peptides in tumor immunopeptidomes. Nat Commun. 2020:11:1293. 10.1038/s41467-020-14968-9. 32157095 PMC7064602

[evag009-B14] Chothani S, Ho L, Schafer S, Rackham O. Discovering microproteins: making the most of ribosome profiling data. RNA Biol. 2023:20:943–954. 10.1080/15476286.2023.2279845. 38013207 PMC10730196

[evag009-B15] Chothani SP, et al A high-resolution map of human RNA translation. Mol Cell. 2022:82:2885–2899.e8. 10.1016/j.molcel.2022.06.023. 35841888

[evag009-B16] Costello JL, et al Predicting the targeting of tail-anchored proteins to subcellular compartments in mammalian cells. J Cell Sci. 2017:130:1675–1687. 10.1242/jcs.200204. 28325759 PMC5450235

[evag009-B17] Couso JP, Patraquim P. Classification and function of small open reading frames. Nat Rev Mol Cell Biol. 2017:18:575–589. 10.1038/nrm.2017.58. 28698598

[evag009-B18] Cuevas MVR, et al Most non-canonical proteins uniquely populate the proteome or immunopeptidome. Cell Rep. 2021:34:108815. 10.1016/j.celrep.2021.108815. 33691108 PMC8040094

[evag009-B19] Davey NE, Haslam NJ, Shields DC, Edwards RJ. SLiMSearch 2.0: biological context for short linear motifs in proteins. Nucleic Acids Res. 2011:39:W56–W60. 10.1093/nar/gkr402. 21622654 PMC3125787

[evag009-B20] Deutsch EW, et al 2024. High-quality peptide evidence for annotating non-canonical open reading frames as human proteins. [preprint]. bioRxiv. 10.1101/2024.09.09.612016.

[evag009-B21] Erdos G, Dosztanyi Z. Analyzing protein disorder with IUPred2A. Curr Protoc Bioinformatics. 2020:70:e99. 10.1002/cpbi.99. 32237272

[evag009-B22] Fredrickson EK, Rosenbaum JC, Locke MN, Milac TI, Gardner RG. Exposed hydrophobicity is a key determinant of nuclear quality control degradation. Mol Biol Cell. 2011:22:2384–2395. 10.1091/mbc.e11-03-0256. 21551067 PMC3128539

[evag009-B23] Frith MC, et al The abundance of short proteins in the mammalian proteome. PLoS Genet. 2006:2:e52. 10.1371/journal.pgen.0020052. 16683031 PMC1449894

[evag009-B24] Fry MY, Saladi SM, Cunha A, Clemons WM Jr. Sequence-based features that are determinant for tail-anchored membrane protein sorting in eukaryotes. Traffic. 2021:22:306–318. 10.1111/tra.12809. 34288289 PMC8380732

[evag009-B25] Guna A, Hegde RS. Transmembrane domain recognition during membrane protein biogenesis and quality control. Curr Biol. 2018:28:R498–R511. 10.1016/j.cub.2018.02.004. 29689233

[evag009-B26] Guo CJ, et al Distinct processing of lncRNAs contributes to non-conserved functions in stem cells. Cell. 2020:181:621–636.e22. 10.1016/j.cell.2020.03.006. 32259487

[evag009-B27] Hao Y, et al Smprot: a database of small proteins encoded by annotated coding and non-coding RNA loci. Brief Bioinform. 2018:19:636–643. 10.1093/bib/bbx005. 28137767

[evag009-B28] Harrison PW, et al Ensembl 2024. Nucleic Acids Res. 2024:52:D891–D899. 10.1093/nar/gkad1049. 37953337 PMC10767893

[evag009-B29] Ingolia NT, Ghaemmaghami S, Newman JRS, Weissman JS. Genome-wide analysis in vivo of translation with nucleotide resolution using ribosome profiling. Science. 2009:324:218–223. 10.1126/science.1168978. 19213877 PMC2746483

[evag009-B30] Jacob E, Unger R. A tale of two tails: why are terminal residues of proteins exposed? Bioinformatics. 2007:23:e225-230. 10.1093/bioinformatics/btl318. 17237096

[evag009-B31] Ji Z, Song R, Regev A, Struhl K. Many lncRNAs, 5′UTRs, and pseudogenes are translated and some are likely to express functional proteins. Elife. 2015:4:e08890. 10.7554/eLife.08890. 26687005 PMC4739776

[evag009-B32] Johansen T, Lamark T. Selective autophagy: ATG8 family proteins, LIR motifs and cargo receptors. J Mol Biol. 2020:432:80–103. 10.1016/j.jmb.2019.07.016. 31310766

[evag009-B33] Kesner JS, et al Noncoding translation mitigation. Nature. 2023:617:395–402. 10.1038/s41586-023-05946-4. 37046090 PMC10560126

[evag009-B34] Kim JM, et al Formyl-methionine as an N-degron of a eukaryotic N-end rule pathway. Science. 2018:362:1019. –+. 10.1126/science.aat0174. PMC6551516 30409808

[evag009-B35] Kim MS, et al A draft map of the human proteome. Nature. 2014:509:575–581. 10.1038/nature13302. 24870542 PMC4403737

[evag009-B36] Kirchner P, et al Proteome-wide analysis of chaperone-mediated autophagy targeting motifs. PLoS Biol. 2019:17:e3000301. 10.1371/journal.pbio.3000301. 31150375 PMC6561683

[evag009-B37] Koren I, et al The eukaryotic proteome is shaped by E3 ubiquitin ligases targeting C-terminal degrons. Cell. 2018:173:1622–1635.e14. 10.1016/j.cell.2018.04.028. 29779948 PMC6003881

[evag009-B38] Kyte J, Doolittle RF. A simple method for displaying the hydropathic character of a protein. J Mol Biol. 1982:157:105–132. 10.1016/0022-2836(82)90515-0. 7108955

[evag009-B39] Ladoukakis E, Pereira V, Magny EG, Eyre-Walker A, Couso JP. Hundreds of putatively functional small open reading frames in *Drosophila*. Genome Biol. 2011:12:R118. 10.1186/gb-2011-12-11-r118. 22118156 PMC3334604

[evag009-B40] Lamark T, Johansen T. Mechanisms of selective autophagy. Annu Rev Cell Dev Biol. 2021:37:143–169. 10.1146/annurev-cellbio-120219-035530. 34152791

[evag009-B41] Laumont CM, et al Noncoding regions are the main source of targetable tumor-specific antigens. Sci Transl Med. 2018:10:eaau5516. 10.1126/scitranslmed.aau5516. 30518613

[evag009-B42] Loughran G, et al Unusually efficient CUG initiation of an overlapping reading frame in POLG mRNA yields novel protein POLGARF. Proc Natl Acad Sci U S A. 2020:117:24936–24946. 10.1073/pnas.2001433117. 32958672 PMC7547235

[evag009-B43] Lu SH, et al A hidden human proteome encoded by ‘non-coding’ genes. Nucleic Acids Res. 2019:47:8111–8125. 10.1093/nar/gkz646. 31340039 PMC6735797

[evag009-B44] Mackowiak SD, et al Extensive identification and analysis of conserved small ORFs in animals. Genome Biol. 2015:16:179. 10.1186/s13059-015-0742-x. 26364619 PMC4568590

[evag009-B45] MacLennan DH, Kranias EG. Phospholamban: a crucial regulator of cardiac contractility. Nat Rev Mol Cell Biol. 2003:4:566–577. 10.1038/nrm1151. 12838339

[evag009-B46] Makaros Y, et al Ubiquitin-independent proteasomal degradation driven by C-degron pathways. Mol Cell. 2023:83:1921–1935.e7. 10.1016/j.molcel.2023.04.023. 37201526 PMC10237035

[evag009-B47] Martinez TF, et al Accurate annotation of human protein-coding small open Reading frames. Nat Chem Biol. 2020:16:458–468. 10.1038/s41589-019-0425-0. 31819274 PMC7085969

[evag009-B48] Mas-Ponte D, et al LncATLAS database for subcellular localization of long noncoding RNAs. RNA. 2017:23:1080–1087. 10.1261/rna.060814.117. 28386015 PMC5473142

[evag009-B49] Mock JY, et al Bag6 complex contains a minimal tail-anchor-targeting module and a mock BAG domain. Proc Natl Acad Sci U S A. 2015:112:106–111. 10.1073/pnas.1402745112. 25535373 PMC4291651

[evag009-B50] Monsellier E, Chiti F. Prevention of amyloid-like aggregation as a driving force of protein evolution. EMBO Rep. 2007:8:737–742. 10.1038/sj.embor.7401034. 17668004 PMC1978086

[evag009-B51] Morales J, et al A joint NCBI and EMBL-EBI transcript set for clinical genomics and research. Nature. 2022:604:310–315. 10.1038/s41586-022-04558-8. 35388217 PMC9007741

[evag009-B52] Mudge JM, et al Standardized annotation of translated open reading frames. Nat Biotechnol. 2022:40:994–999. 10.1038/s41587-022-01369-0. 35831657 PMC9757701

[evag009-B53] Nelson BR, et al A peptide encoded by a transcript annotated as long noncoding RNA enhances SERCA activity in muscle. Science. 2016:351:271–275. 10.1126/science.aad4076. 26816378 PMC4892890

[evag009-B54] Odermatt A, et al Characterization of the gene encoding human sarcolipin (SLN), a proteolipid associated with SERCA1: absence of structural mutations in five patients with Brody disease. Genomics. 1997:45:541–553. 10.1006/geno.1997.4967. 9367679

[evag009-B55] Ouspenskaia T, et al Unannotated proteins expand the MHC-I-restricted immunopeptidome in cancer. Nat Biotechnol. 2022:40:209–217. 10.1038/s41587-021-01021-3. 34663921 PMC10198624

[evag009-B56] Prensner JR, et al Noncanonical open reading frames encode functional proteins essential for cancer cell survival. Nat Biotechnol. 2021:39:697–704. 10.1038/s41587-020-00806-2. 33510483 PMC8195866

[evag009-B57] Prensner JR, et al What can Ribo-seq and proteomics tell us about the non-canonical proteome? Mol Cell Proteomics. 2023:22:100631. 10.1016/j.mcpro.2023.100631.. PMC10506109 37572790

[evag009-B58] Prieto G, Fullaondo A, Rodriguez JA. Prediction of nuclear export signals using weighted regular expressions (Wregex). Bioinformatics. 2014:30:1220–1227. 10.1093/bioinformatics/btu016. 24413524

[evag009-B59] Pruitt KD, et al The consensus coding sequence (CCDS) project: identifying a common protein-coding gene set for the human and mouse genomes. Genome Res. 2009:19:1316–1323. 10.1101/gr.080531.108. 19498102 PMC2704439

[evag009-B60] Quinlan AR, Hall IM. BEDTools: a flexible suite of utilities for comparing genomic features. Bioinformatics. 2010:26:841–842. 10.1093/bioinformatics/btq033. 20110278 PMC2832824

[evag009-B61] Raj A, et al Thousands of novel translated open reading frames in humans inferred by ribosome footprint profiling. Elife. 2016:5:e13328. 10.7554/eLife.13328. 27232982 PMC4940163

[evag009-B62] R Core Team . R: a language and environment for statistical computing. R Foundation for Statistical Computing; 2021.

[evag009-B63] Rice P, Longden I, Bleasby A. EMBOSS: the European molecular biology open software suite. Trends Genet. 2000:16:276–277. 10.1016/S0168-9525(00)02024-2. 10827456

[evag009-B64] Ruiz-Orera J, Alba MM. Translation of small open Reading frames: roles in regulation and evolutionary innovation. Trends Genet. 2019:35:186–198. 10.1016/j.tig.2018.12.003. 30606460

[evag009-B65] Salovska B, et al Isoform-resolved correlation analysis between mRNA abundance regulation and protein level degradation. Mol Syst Biol. 2020:16:e9170. 10.15252/msb.20199170. 32175694 PMC7073818

[evag009-B66] Samandi S, et al Deep transcriptome annotation enables the discovery and functional characterization of cryptic small proteins. Elife. 2017:6:e27860. 10.7554/eLife.27860. 29083303 PMC5703645

[evag009-B67] Sandmann CL, et al Evolutionary origins and interactomes of human, young microproteins and small peptides translated from short open reading frames. Mol Cell. 2023:83:994–1011.e18. 10.1016/j.molcel.2023.01.023. 36806354 PMC10032668

[evag009-B68] Schlesinger D, et al A large-scale sORF screen identifies putative microproteins involved in cancer cell fitness. iScience. 2025:28:111884. 10.1016/j.isci.2025.111884. 40124493 PMC11929002

[evag009-B69] Schnebert S, et al Diving into the evolutionary history of HSC70-linked selective autophagy pathways: endosomal microautophagy and chaperone-mediated autophagy. Cells. 2022:11: 1945. 10.3390/cells11121945. 35741074 PMC9221867

[evag009-B70] Schubert U, et al Rapid degradation of a large fraction of newly synthesized proteins by proteasomes. Nature. 2000:404:770–774. 10.1038/35008096. 10783891

[evag009-B71] Shao Y, et al GenTree, an integrated resource for analyzing the evolution and function of primate-specific coding genes. Genome Res. 2019:29:682–696. 10.1101/gr.238733.118. 30862647 PMC6442393

[evag009-B72] Slavoff SA, et al Peptidomic discovery of short open reading frame-encoded peptides in human cells. Nat Chem Biol. 2013:9:59–64. 10.1038/nchembio.1120. 23160002 PMC3625679

[evag009-B73] Soto C, Pritzkow S. Protein misfolding, aggregation, and conformational strains in neurodegenerative diseases. Nat Neurosci. 2018:21:1332–1340. 10.1038/s41593-018-0235-9. 30250260 PMC6432913

[evag009-B74] Stein CS, et al Mitoregulin: a lncRNA-encoded microprotein that supports mitochondrial supercomplexes and respiratory efficiency. Cell Rep. 2018:23:3710. –+. 10.1016/j.celrep.2018.06.002. 29949756 PMC6091870

[evag009-B75] Tapial J, et al An atlas of alternative splicing profiles and functional associations reveals new regulatory programs and genes that simultaneously express multiple major isoforms. Genome Res. 2017:27:1759–1768. 10.1101/gr.220962.117. 28855263 PMC5630039

[evag009-B76] Timms RT, et al A glycine-specific N-degron pathway mediates the quality control of protein N-myristoylation. Science. 2019:365:aaw4912. 10.1126/science.aaw4912. PMC7090375 31273098

[evag009-B77] Timms RT, Koren I. Tying up loose ends: the N-degron and C-degron pathways of protein degradation. Biochem Soc Trans. 2020:48:1557–1567. 10.1042/BST20191094. 32627813 PMC7458402

[evag009-B78] Tong G, Hah N, Martinez TF. Comparison of software packages for detecting unannotated translated small open reading frames by Ribo-seq. Brief Bioinform. 2024:25:bbae268. 10.1093/bib/bbae268. 38842510 PMC11155197

[evag009-B79] Tsirigos KD, Peters C, Shu N, Kall L, Elofsson A. The TOPCONS web server for consensus prediction of membrane protein topology and signal peptides. Nucleic Acids Res. 2015:43:W401–W407. 10.1093/nar/gkv485. 25969446 PMC4489233

[evag009-B80] Vakirlis N, Vance Z, Duggan KM, McLysaght A. De novo birth of functional microproteins in the human lineage. Cell Rep. 2022:41:111808. 10.1016/j.celrep.2022.111808. 36543139 PMC10073203

[evag009-B81] van der Lee R, et al Intrinsically disordered segments affect protein half-life in the cell and during evolution. Cell Rep. 2014:8:1832–1844. 10.1016/j.celrep.2014.07.055. 25220455 PMC4358326

[evag009-B82] Vanderperre B, et al Direct detection of alternative open reading frames translation products in human significantly expands the proteome. PLoS One. 2013:8:e70698. 10.1371/journal.pone.0070698. 23950983 PMC3741303

[evag009-B83] van Heesch S, et al The translational landscape of the human heart. Cell. 2019:178:242–260.e29. 10.1016/j.cell.2019.05.010. 31155234

[evag009-B84] Varshavsky A . N-degron and C-degron pathways of protein degradation. Proc Natl Acad Sci U S A. 2019:116:358–366. 10.1073/pnas.1816596116. 30622213 PMC6329975

[evag009-B85] Volders PJ, et al LNCipedia 5: towards a reference set of human long non-coding RNAs. Nucleic Acids Res. 2019:47:D135–D139. 10.1093/nar/gky1031. 30371849 PMC6323963

[evag009-B86] Wacholder A, et al Community benchmarking and evaluation of human unannotated microprotein detection by mass spectrometry based proteomics. Nat Commun. 2026. 10.1038/s41467-025-68002-x 2025. PMC12865046 41559053

[evag009-B87] Wacholder A, Carvunis AR. Biological factors and statistical limitations prevent detection of most noncanonical proteins by mass spectrometry. PLoS Biol. 2023:21:e3002409. 10.1371/journal.pbio.3002409. 38048358 PMC10721188

[evag009-B88] Walsh I, Seno F, Tosatto SC, Trovato A. PASTA 2.0: an improved server for protein aggregation prediction. Nucleic Acids Res. 2014:42:W301–W307. 10.1093/nar/gku399. 24848016 PMC4086119

[evag009-B89] Yang H, Li Q, Stroup EK, Wang S, Ji Z. Widespread stable noncanonical peptides identified by integrated analyses of ribosome profiling and ORF features. Nat Commun. 2024:15:1932. 10.1038/s41467-024-46240-9. 38431639 PMC10908861

[evag009-B90] Yewdell JW, Anton LC, Bennink JR. Defective ribosomal products (DRiPs)—a major source of antigenic peptides for MHC class I molecules? J Immunol. 1996:157:1823–1826. 10.4049/jimmunol.157.5.1823. 8757297

[evag009-B91] Yim WW, Mizushima N. Lysosome biology in autophagy. Cell Discov. 2020:6:6. 10.1038/s41421-020-0141-7. 32047650 PMC7010707

[evag009-B92] Zecha J, et al Peptide level turnover measurements enable the study of proteoform dynamics. Mol Cell Proteomics. 2018:17:974–992. 10.1074/mcp.RA118.000583. 29414762 PMC5930408

[evag009-B93] Zhang P, et al Genome-wide identification and differential analysis of translational initiation. Nat Commun. 2017:8:1749. 10.1038/s41467-017-01981-8. 29170441 PMC5701008

[evag009-B94] Zheng C, et al CRISPR-Cas9-based functional interrogation of unconventional translatome reveals human cancer dependency on cryptic non-canonical open Reading frames. Nat Struct Mol Biol. 2023:30:1878–1892. 10.1038/s41594-023-01117-1. 37932451 PMC10716047

