# Supplementary material for: Degradation Determinants Are Abundant in Human Noncanonical Proteins and Minor Annotated Isoforms
Source: Genome Biol Evol. 2026 Jan 19;18(2):evag009. doi: 10.1093/gbe/evag009 (PMC12863090; doi:10.1093/gbe/evag009)
Supplement: evag009_Supplementary_Data [file evag009_supplementary_data.zip › Supplementary Material.pdf]

Supplementary Material

Degradation determinants are abundant in human noncanonical proteins and minor annotated isoforms

Claudio Casola[a,b,c,1], Adekola Owoyemi[a] and Nikolaos Vakirlis[d,1]

[a]Department of Ecology and Conservation Biology, Texas A&M University, College Station, TX 77845

[b]Interdisciplinary Doctoral Degree Program in Ecology and Evolutionary Biology, Texas A&M University, College Station, TX 77845

[c]Interdisciplinary Doctoral Degree Program in Genetics and Genomics, Texas A&M University, College Station, TX 77845

[d]Hellenic Pasteur Institute, Athens 11521, Greece

Corresponding Authors Information:

[1]Claudio Casola: 534 John Kimbrough Blvd, TAMU 2258, College Station, TX 77843-2258, 979-845-8803, ccasola@tamu.edu

[1]Nikolaos Vakirlis: 127 Vas. Sofias Ave, 115 21, Athens, Greece,

+302106478524 , n.vakirlis@pasteur.gr

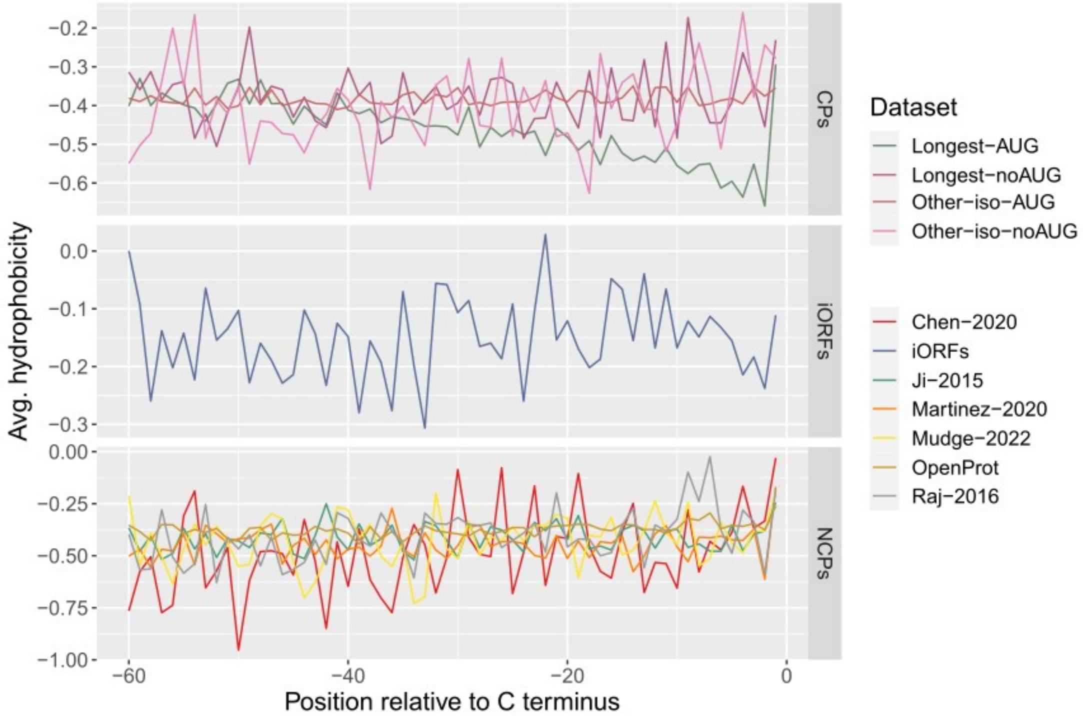

Figure S1. Average hydrophobicity (Kyte-Doolittle scale) in the last 60 amino acids of CPs, NCPs and iORFs.

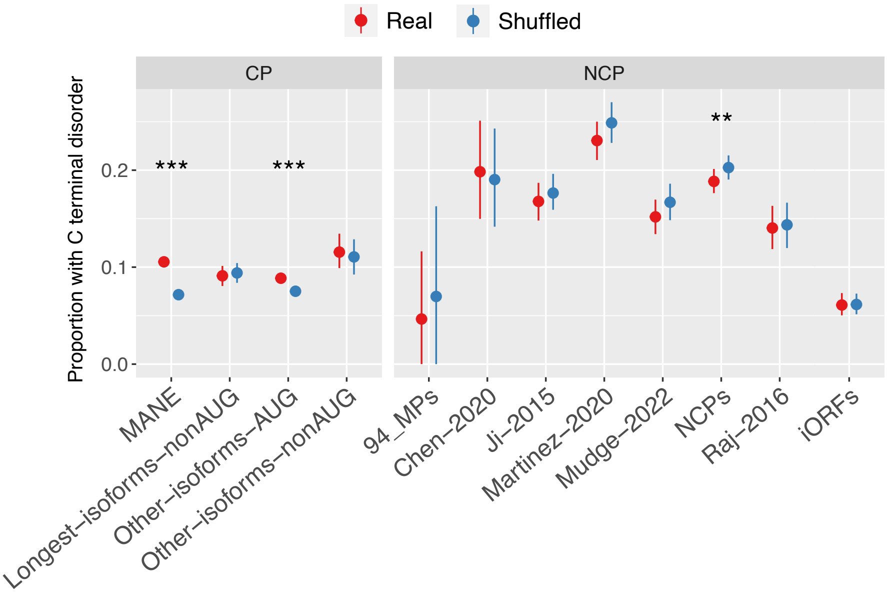

Figure S2. Proportion of sequences with C-terminal intrinsically disordered regions.

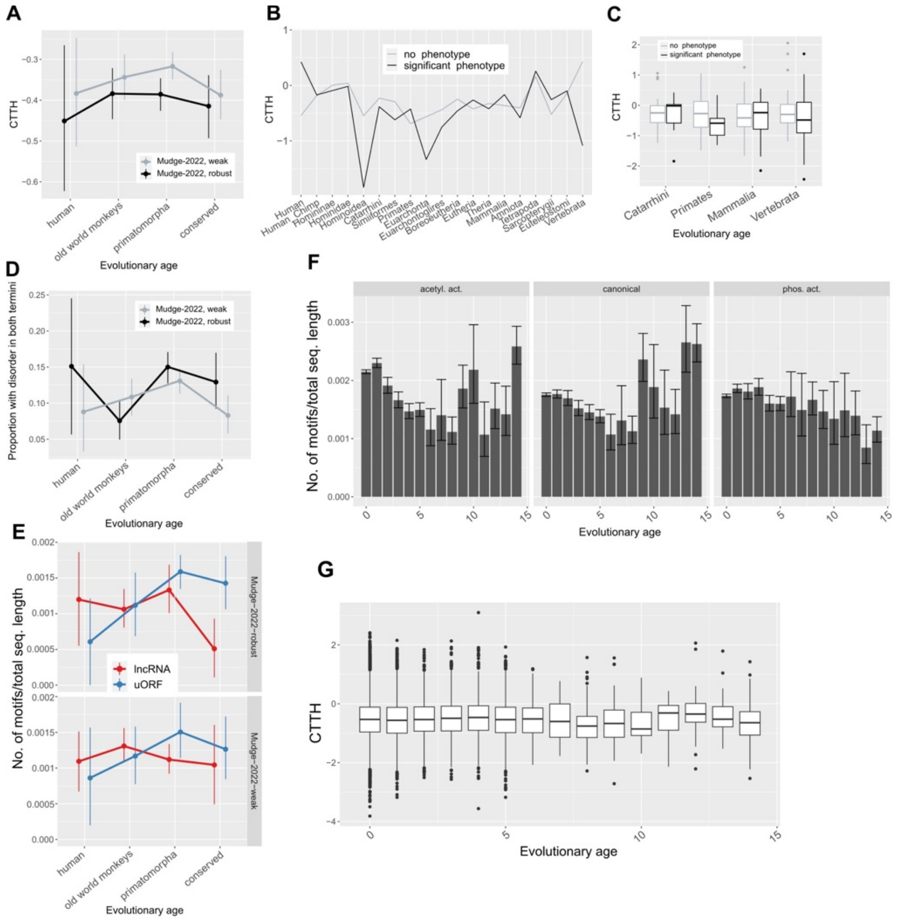

Figure S3. (A) Average CTTH in noncanonical proteins with increasing age from left to right as assessed by Sandmann et al. (2023) on data from Mudge et al. (2022) (weak and robust datasets). **(B)** Average CTTH in NCPs with increasing age from left to right as assessed by Vakirlis et al. (2022) on data from (Chen, et al. 2020). Microproteins with significant phenotypes and no phenotypes as measured by Chen et al. are shown separately. **(C)** Same as B but on four broader groups of evolutionary age. **(D)** Frequency of KFERQ-like motifs in NCP types from the robust and weak datasets from Mudge et al. (2022)., with increasing age from left to right as assessed by Sandmann et al. (2023). **(E)** Frequency of IDRs on both termini in NCPs assessed by Sandmann et al. (2023) on the robust and weak datasets from Mudge et al. (2022). **(F)** Frequency of KFERQ-like motifs of different types, in MANE isoforms with decreasing evolutionary age as assessed by Shao et al. (2019). **(G)** Distribution of CTTH values in MANE isoforms with decreasing evolutionary age as assessed by Shao et al. (2019).

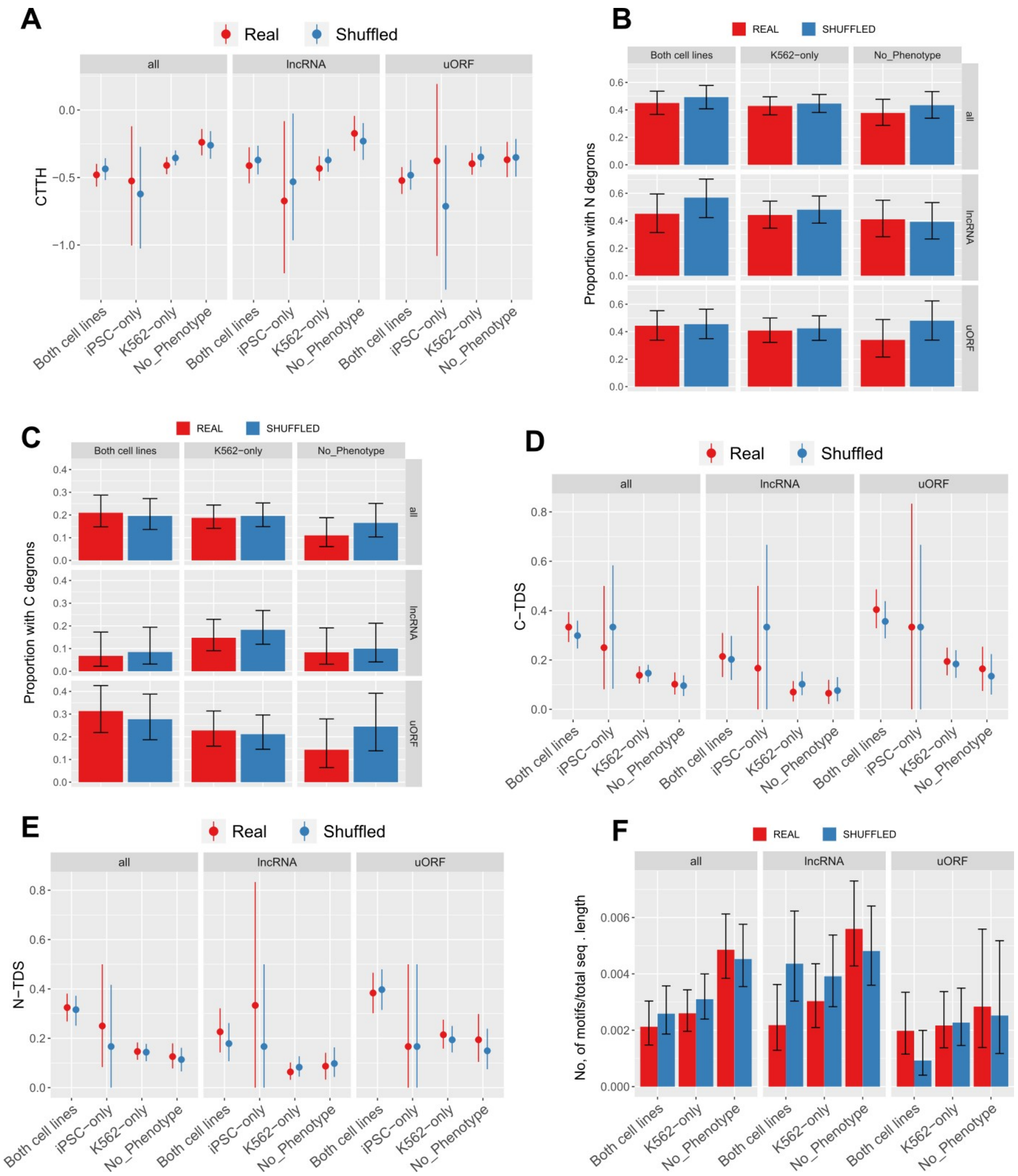

Figure S4. Degradation determinants in noncanonical proteins tested in K562 and iPSC cell lines. **(A)** CTTH. **(B-C)** Degrons. **(D-F)** IDRs. **(G)** KFERQ-like motifs frequency. K562: proteins with phenotype in K562 cells. Both: proteins with phenotype in K562 and iPSC cells. NoPh: proteins with no phenotype.

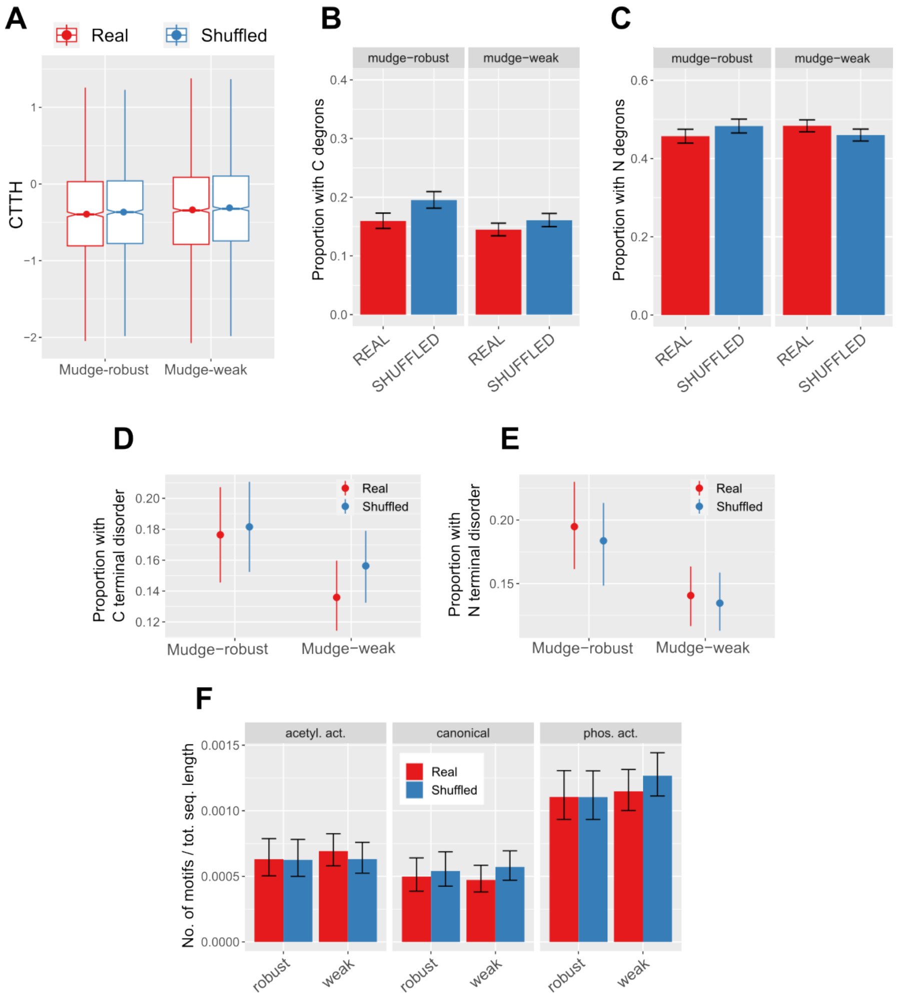

Figure S5. Degradation determinants in noncanonical proteins expressed in one study (weak) or multiple studies (robust). **(A)** CTTH. **(B-C)** Degrons **(D-F)** IDRs. **(G)** KFERQ-like motifs frequency.
